# The *transformer* gene controls sexual development in *Drosophila suzukii*

**DOI:** 10.1111/1744-7917.70031

**Published:** 2025-03-30

**Authors:** Ying Yan, Jing Zhao, Jonas Schwirz, Cristina Borghesi, Conghui Liu, Bo Liu, Wanqiang Qian, Fanghao Wan, Marc F. Schetelig

**Affiliations:** ^1^ Department of Insect Biotechnology in Plant Protection Justus‐Liebig‐University Gießen, Institute for Insect Biotechnology Giessen Germany; ^2^ Shenzhen Branch, Guangdong Laboratory for Lingnan Modern Agriculture, Genome Analysis Laboratory of the Ministry of Agriculture and Rural Affairs of the People's Republic of China, Agricultural Genomics Institute at Shenzhen Chinese Academy of Agricultural Sciences Shenzhen China

**Keywords:** courtship behavior, gene splicing, pigmentation, sex determination, spotted‐wing *Drosophila*, Tetracycline‐Off system

## Abstract

The genetic network of sex determination in the model organism *Drosophila melanogaster* was investigated in great detail. Such knowledge not only advances our understanding of the evolution and regulation of sexual dimorphism in insects, but also serves as a basis for developing genetic control strategies for pest species. In this study, we isolated the sex determination gene *transformer* (*Dstra*) from a global fruit pest, the spotted‐wing *Drosophila* (*Drosophila suzukii*), and characterized its gene organization. By comparing the deduced protein sequence of Dstra with its orthologs from 22 species, we found that *tra* genes from Dipteran species are divergent. Our research demonstrated that *Dstra* undergoes sex‐specific splicing, and we validated its developmental expression profile. We engineered a *piggyBac‐*based transformation vector expressing the complete *Dstra* coding sequence under the control of the Tetracycline‐Off system. Through germ‐line transformation, we generated 4 independent transgenic lines, producing pseudo‐females from chromosomal males in the absence of tetracycline. This observation indicated ectopic expression of *Dstra*, confirmed by the detection of female *Dstra* transcripts in transgenic males. The pseudo‐females exhibited altered wing patterns, feminized abdomen, abnormal reproductive organs, and disrupted sexual behavior. Ectopic expression of *Dstra* affected the sex‐specific splicing pattern of the downstream gene *fruitless*, but not *doublesex*. In conclusion, our study provides comprehensive genetic, morphological, and behavioral evidence that *Dstra* controls sexual development in *D. suzukii*. We discuss the potential applications of this research for genetic control strategies targeting *Dstra* or using its gene elements.

## Graphical abstract

The transformer gene plays a key role in the genetic pathway for sexual development in Drosophila suzukii

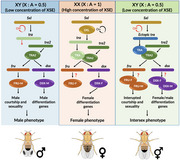

## Introduction

Sexual development, one of the most important developmental processes in insects, determines the fundamental differences between males and females. In the model organism *Drosophila melanogaster*, the genetic basis of sex determination was studied and illustrated in great detail (Cline & Meyer, 1996; Penalva & Sanchez, 2003; Sanchez, 2008; Verhulst *et al.*, 2010a; Bopp *et al.*, 2014). Specifically, at the top of the sex determination pathway, the master gene *Sex‐lethal* (*Sxl*) responds to the X:A signal and is only activated in females (2X;2A) but not in males (X;2A). Meanwhile, *male‐specific lethal* (*msl*) genes contribute to a heteromultimeric MSL complex, which activates the dosage compensation in males by hyper‐transcription of the single X chromosome. In females, a key gene (*msl‐2*) for MSL complex is prevented by SXL protein; therefore, X chromosome dosage compensation is suppressed in this sex (Cline & Meyer, 1996; Penalva & Sanchez, 2003). *Sxl* autoregulates its expression and mediates the RNA splicing of the downstream gene *transformer* (*tra*) differently in males and females. Only the female form of *tra* RNA codes a functional protein interacting with the product from *transformer 2* (*tra2*). The TRA/TRA2 complex switches *doublesex* (*dsx*) and *fruitless* (*fru*) into the female modes of splicing, and the corresponding products, DSX and FRU, ultimately control most aspects of sexual development and behavior (Black, 2003; Pomiankowski *et al.*, 2004; Sanchez, 2008).

The role of *tra* in the sex determination pathway is believed to be conserved in many insect species (Shearman, 2002; Sanchez, 2008; Verhulst *et al.*, 2010a; Gempe & Beye, 2011; Saccone, 2022). For example, both *tra* and *dsx* genes have been identified from the family Apidae and Pteromalidae of Hymenoptera as well as the family Tephritoidae, Drosophilidae, Muscidae, and Calliphoridae of Diptera (Verhulst *et al.*, 2010a). In *Tribolium castaneum* (Coleoptera: Tenebrionidae), a *tra*‐like feminizing gene has been found that is sex‐specifically spliced and shows conserved domains as in some *tra* orthologs from Diptera species (Shukla & Palli, 2012). RNA silencing of *tra* led to female‐to‐male sex transformation in tephritid species such as *Anastrepha ludens*, *A. suspensa, Bactrocera dorsalis*, *B. oleae*, *Ceratitis capitata* (Pane *et al.*, 2002; Lagos *et al.*, 2007; Schetelig *et al.*, 2012; Liu *et al.*, 2015), as well as in calliphorid species such as *Lucilia cuprina*, *Cochliomyia hominivorax*, *C. macellaria*, and *L. sericata* (Concha & Scott, 2009; Li *et al.*, 2013). While the downstream pathway of *tra*, involving sex‐specific regulations of *dsx* and *fru*, is generally conserved, different upstream elements were identified in different insect orders and considered as instructive signals for the *tra* establishment including X:A signal, M factors, maternal imprint, and complementary sex determination (csd) genes (Verhulst *et al.*, 2010b; Bopp *et al.*, 2014; Sharma *et al.*, 2017). Such variations at the top of the sex determination cascade highlight the evolution of insect sex‐determining systems (Shearman, 2002; Sanchez, 2008; Bopp *et al.*, 2014).

Exploring the sex determination mechanisms in insects could facilitate the development of pest control approaches (Koukidou & Alphey, 2014; Lutrat *et al.*, 2019; Yan *et al.*, 2024). For example, genetic control strategies such as the female‐specific Release of Insects carrying a Dominant Lethal (fs‐RIDL) (Fu *et al.*, 2007; Li *et al.*, 2014) and Transgenic Embryonic Sexing Systems (TESS) (Schetelig & Handler, 2012; Yan & Scott, 2015; Schetelig *et al.* 2016; Yan *et al.* 2020b) rely on the female‐specifically spliced intron from *tra* genes (traF). In these strategies, traF intron was engineered into a lethal effector gene; therefore, only females produce functional products and die. Instead of killing females, a sex ratio distortion (SRD) strategy was suggested by targeting feminizing gene(s) (Saccone *et al.*, 2007; Williamson *et al.*, 2021) or ectopic expression of the M factor (Adelman & Tu, 2016; Meccariello *et al.*, 2019) to transform females into males. Some state‐of‐the‐art clustered regularly interspaced short palindromic repeats (CRISPR)‐based control strategies, including gene drive, CRISPR‐engineered genetic sexing strains, and precision‐guided sterile insect technique (pgSIT), were also demonstrated by targeting the sex determination genes (Yan *et al.*, 2023).

The spotted‐wing *Drosophila* (*Drosophila suzukii*; Diptera, Drosophilidae) is an invasive pest threatening fruit production worldwide (Rogers *et al.*, 2016; Dos Santos *et al.*, 2017) that is named after the black spots on the male wings. The females possess a serrated ovipositor, which enables them to penetrate the skin of many soft‐skinned fruits of economic importance (Rota‐Stabelli *et al.*, 2013; Atallah *et al.*, 2014). In this study, we isolated the *tra* gene from *D. suzukii* (*Dstra*), and its genomic organization was identified. We have documented the developmental expression profile of *Dstra*, which undergoes sex‐specific splicing. Transgenic *D. suzukii* lines expressing *Dstra* under the control of the Tetracycline (Tet)‐off system were generated and used to study the functions of *Dstra* in sexual development. We discuss the results of sex reversion in *D. suzukii* and potential genetic control methods using this gene.

## Materials and methods

### Insect rearing and germ‐line transformation

The wild‐type (WT) USA strain and transgenic *D. suzukii* lines were maintained at 25 °C and 55%–60% humidity. The WT‐USA strain and an X‐linked V84_F15f1 strain (not published) were reared on a tetracycline‐free diet, whereas the V262 strains were maintained on the same diet supplemented with 100 *µ*g/mL tetracycline (Tet‐diet; Thermo Fisher Scientific). The germ‐line transformation was carried out as previously reported (Schwirz *et al.*, 2020; Schetelig *et al.*, 2021). Briefly, eggs were collected from the WT‐USA strain maintained on Tet‐diet for at least 2 d before the injection. The eggs were desiccated for 6–10 min and overlaid with halocarbon oil 700 (Sigma‐Aldrich). A mixture of the *piggyBac* donor construct (700 ng/*µ*L) and the *phsp‐pBac* transposase plasmid (300 ng/*µ*L) or *in vitro* synthesized *piggyBac* RNA helper (300 ng/*µ*L) was injected into WT embryos. Surviving G_0_ adults were backcrossed to WT males or virgin females with putative G_1_ transformant progeny selected by fluorescence. Segregation tests were conducted by outcrossing the transformants to WT flies. Independent homozygous strains were established by screening the fluorescence intensity at the 3rd‐instar larval stage for homozygous individuals.

### Gene sequence isolation and analysis

This project was initiated in 2014 when the gene query function was not available in SWDbase (Chiu *et al.*, 2013). Therefore, the *Dstra* gene and messenger RNA (mRNA) sequences were identified by BLAST search in the *D. suzukii* genome and transcriptome data (https://spottedwingflybase.org/) using *Dmtra* gene (FBgn0003741) and mRNA sequences (FBtr0075364), which were obtained from FlyBase (https://flybase.org/) as queries, respectively. Based on the hits recovered for each search, primers (Table. S1) were designed to amplify the genomic sequence of *Dstra* from the WT‐USA strain, as well as mRNA sequences from complementary DNA (cDNA) pools of both sexes. A 1 736 bp product of the *Dstra* gene was amplified from embryonic genomic DNA using primers P86 and P88, and a 766 bp male transcript was amplified from male cDNA using primers P85 and P87. The polymerase chain reaction (PCR) products were separated by 1% agarose gel electrophoresis and extracted from the gels using the QIAquick Gel Extraction Kit (Qiagen) before cloning in vector pCR4‐TOPO. The presence of inserts was confirmed by restriction digestion with EcoRI and sequencing using M13 primers. Similarly, a pCR4 plasmid containing the female transcript (603 bp) was generated earlier in order to obtain the nuclear localization signal (NLS) from *Dstra* (Schwirz *et al.*, 2020). The 576 bp coding region sequence (CDS) was disclosed based on the female transcript using Geneious software, and the exon and intron regions were identified by comparing the male or female transcript to the genomic sequence. The genomic DNA was prepared from *D. suzukii* embryos using DNAzol Reagent (Thermo Fisher Scientific), and the total RNA was isolated from male or female flies using the ZR Tissue & Insect RNA MicroPrep kit (Zymo Research, USA) and treated with Turbo DNase (Thermo Fisher Scientific, USA). One microgram RNA was used to synthesize cDNA using the iScript cDNA Synthesis Kit (Bio‐Rad, USA). The PCR was assembled in 25 *µ*L reactions comprising 0.1 *µ*L Platinum Taq DNA polymerase (Invitrogen, USA), 2.5 *µ*L of a 10× PCR buffer, 1 *µ*L of 50 mmol/L MgCl_2_, 2.5 *µ*L of 10 mmol/L deoxynucleotide triphosphates (dNTPs), 0.75 *µ*L of 10 *µ*mol/L of each primer, and 1 *µ*L diluted genomic DNA (10–25 ng/*µ*L) or cDNA (1 : 5 dilution). The cycling conditions were 95 °C for 2 min, followed by 35 cycles of 95 °C for 30 s, 52 °C for 30 s, and 72 °C for 60 s (P85/ P87) or 90 s (P86/ P88), and a final extension step at 72 °C for 5 min. Sequence translation, multiple sequence alignment, and phylogenetic analysis were performed using the Geneious Prime software. The MEME analysis for conserved motifs was performed on https://meme‐suite.org/meme/tools/meme. The search was set to find 15 motifs, with a size range of 6 to 50 bp.

### Plasmid construction

The inverted repeats were constructed using the pGEM_WIZ vector as previously described (Bao & Cagan, 2006; Williamson *et al.*, 2021). Specifically, 2 sets of primers were designed to amplify the forward and reverse fragments of *Dstra* female‐specific coding region sequence. PCR using the first primer set (P692 and P694) had XhoI‐MluI and AvrII restriction sites added to the 5ʹ and 3ʹ of the forward sequence, respectively. PCR using the second primer set (P695 and P696) had HindIII‐BsmBI and NheI restriction sites added to the 5ʹ and 3ʹ of the reverse sequence, respectively. The product of the forward sequence was digested with XhoI and AvrII and cloned into *pGEM_WIZ* cut with the same enzymes. The resulting plasmid *pGEM_WIZ_Dstra‐CDS* was digested with HindIII and NheI and ligated to the product of the reverse sequence cut with the same enzymes to obtain *pGEM_WIZ_Dstra‐CDS_IR*. Then the *Dstra‐CDS_IR* was excised out and inserted into *V229_ pBXLII_attP_PUbAmCyan_Dsnullo‐tTA‐SV40_TREhs43‐DsHid^Ala4^‐CctraF‐SV40* (Schetelig *et al.*, 2021) using MluI and BsmBI restriction enzymes, to generate *V262_pBXLII_attP_PUbAmCyan_Dsnullo‐tTA_TREhs43‐Dstra‐CDS‐IR‐SV40*.

### Reverse‐transcriptase (RT)‐PCR and genomic PCR

The sex‐specific transcripts of the *Dstra* gene were determined by RT‐PCR using 3 sets of primers (P742 paired with P703, P704, P705). The expression profiles of *Dstra*, a male‐specific gene *Dsβ2t* (*β2‐tubulin*) (Yan *et al.*, 2021b), a female‐specific gene *DsYp1* (Yolk protein 1) (Li & Handler, 2017), and the housekeeping gene *DsTBP* (TATA‐binding protein) (Zhai *et al.*, 2014) were determined by RT‐PCR using the primers listed in Table S1. The eggs were frozen in liquid nitrogen at the desired developmental time point. The larvae and pupae were collected directly from stock vials at the desired stage, and adult males and females were isolated immediately after eclosion and sampled 5 d later. For genomic PCR, genomic DNA was extracted from transgenic flies as described above. Three primer pairs (P516/P420; MFS5/P704; P704/MFS10) were used to amplify parts of the V262 transgene construct. Total RNA or genomic DNA was isolated, and cDNA was synthesized as previously described. PCRs to check the alternative splicing of *Dstra* were set up in a 25‐*µ*L reaction containing 5 *µ*L of 5×Q5 Reaction Buffer (New England BioLabs Inc.), 0.5 *µ*L of 10 mmol/L dNTP mix, 1.25 *µ*L of each primer (0.5 *µ*mol/L), 1 *µ*L of diluted cDNA and 0.25 *µ*L of Q5 High‐Fidelity DNA Polymerase (New England BioLabs Inc.). The PCR reaction was subjected to the following thermal cycling parameters: 98 °C for 30 s, followed by 35 cycles of 98 °C for 30 s, 52 °C (P742 with P703, P704 or P705), and 72 °C for 30 s, and then a final extension for 2 min at 72 °C. Other PCRs were assembled using Platinum Taq polymerase as described above, and each reaction was heated to 95 °C for 2 min, followed by 35 cycles of 95 °C for 30 s, 55 °C (for P740/P741, P1358/ P1359, P516/P420, MFS5/P704, P704/MFS10) or 60 °C (for P1453/P1454) for 30 s, and 72 °C for 30 s (for P740/P741, P1358/ P1359, P1453/P1454), 60 s (for P516/P420, MFS5/P704) or 90 s (for P704/MFS10), and then a final extension for 5 min at 72 °C. The products were gel purified as described and sequenced with the corresponding PCR primers. PCR reactions were set up to analyze the alternative splicing of *Dsfru* and *Dsdsx*. Each reaction consisted of 20 *µ*L volume containing 1× Phusion Flash High‐Fidelity PCR Master Mix (Thermo Scientific, F548S) and 500 nmol/L of each primer. The thermal cycling parameters were as follows: initial denaturation at 98 °C for 10 s, followed by 35 cycles of denaturation at 98 °C for 1 s, annealing at 52 °C for 30 s, and extension at 72 °C for 30 s, with a final extension step at 72 °C for 1 min.

### Fly imaging, crossing, screening, and counting

To verify the sex reversal in V262 strains, tetracycline (100 *µ*g/mL) was fed to the parental flies at the larval stage but not afterward. The offspring were also reared on a tetracycline‐free diet, and the adult males and females (4–7 d old) were used to check the corresponding phenotypes. The testes or ovaries were dissected in Ringer's solution (0.120 mmol/L NaCl, 1.5 mmol/L CaCl_2_, 5 mmol/L KCl, pH 7.4) using a Leica M205FA microscope (Leica Microsystems, Wetzlar, Germany). Abdomens or external genitalia were collected similarly without Ringer's solution. Flies and tissues were imaged using the Leica M205FA with brightfield illumination. To test strain V262_M2f1 at heterozygous condition, 10 homozygous virgin females were crossed with 5 males (3–5 d old) from an X‐linked line V84_F15f1_*pBXLII_attP_PUbDsRed_Dssry‐α‐tTA* (unpublished) in a large food vial (tetracycline‐free, 175 mL volume, 50×100 mm). The flies were transferred to a fresh vial (tetracycline‐free) every day for a further 7 d. In the next generation, 1‐ to 3‐d‐old adult flies were screened using the Leica M205FA with a CFP filter for AmCyan (ex. 436/20; em. 480/40) and a TxRed filter for DsRed (ex. 545/30; em. 620/60) to distinguish genetic males from females. The number of spotted males, spotless males, and females were also counted. To test the strain V262_M2f1 at homozygous condition, 5 males and 10 virgin females from the same strain were crossed similarly as described above, and the number of spotted males, spotless males, and females in the next generation were counted. Both heterozygous and homozygous tests were repeated similarly, except the Tet‐diet was used all the time as a control. Three biological replicates were carried out for all experiments here.

### Mating behavior analysis

The experiments to verify the male courtship and copulation successes were conducted as previously described (Yan *et al.*, 2020c). Briefly, a mating pair of flies was gently aspirated into an arena with a 0.7 cm diameter and a depth of 0.25–0.3 cm from a 96‐well plate (Greiner Bio‐One, Kremsmünster, Austria). The mating was monitored and recorded on video for desired time periods using a DinoLite AM7915MZTL digital microscope (DinoLite, New Taipei City, China). Copulation and male courtship behavior (CB) in the video files were analyzed using Dinocapture software (DinoLite). The criteria for male CB and the calculation for courtship index were previously described (Revadi *et al.*, 2015; Xiao *et al.*, 2017; Yan *et al.*, 2020c). Specifically, the sexual activities of fly pairs were sampled once every 10 s during the first 5 min and for 60 s at minutes 15, 30, 60, 90, 120, 150, 180, 210, 240, and 270 of the recordings. During each sampling period, a score of 1 or 0 was assigned to each pair of flies, indicating the presence or absence of CB. Clearly observable courtship activities of males included orientation, female following, and wing extension. Due to the phenotypic difference between the transgenic spotless males and WT spotted‐wing males, it was possible to determine the CB for each male in these pairs. In contrast, for the pairs using only WT males, a score of 1 was assigned every time when either male showed CB and the average CI was calculated for a single WT male.

### Statistics

Statistical analysis was carried out using SigmaPlot (v14.0). Differences in the fly numbers of each phenotype were analyzed by one‐way analysis of variance (ANOVA) and means (some are square root transformed) were separated using Duncan's Method. The differences in copulation success and CI were analyzed using Chi‐square test.

## Results

### D. suzukii transformer gene is sex‐specifically spliced

**Fig. 1:**
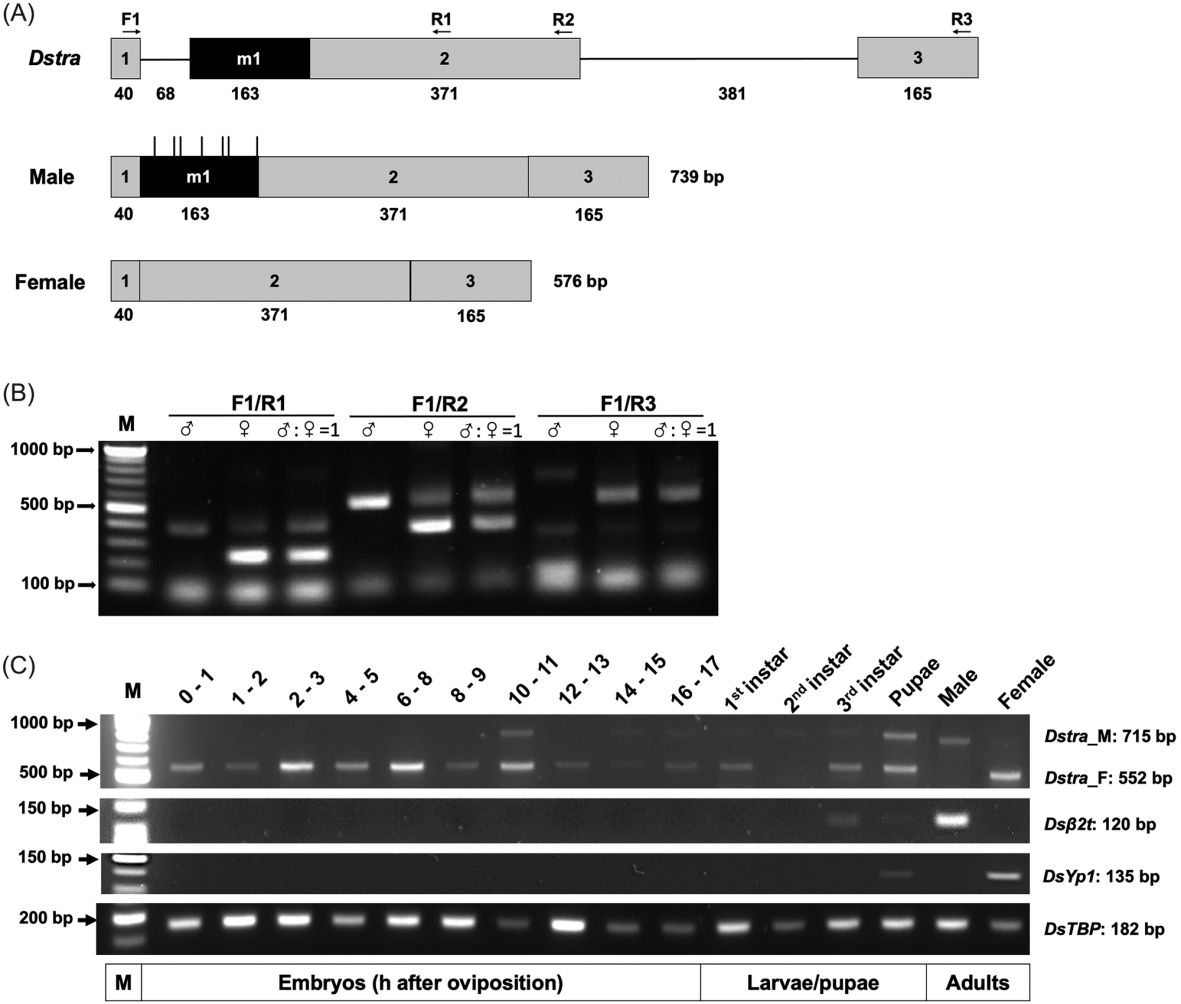
*Drosophila suzukii transformer (Dstra)* gene organization and sex‐specific transcripts. (A) The *Dstra* gene sequence was confirmed by comparing male and female messenger RNA (mRNA) to genomic DNA. *Dstra* consists of 3 exons (gray boxes) and 1 male‐specific exon (black box). Introns are shown as black lines. All exons and introns are drawn to scale, with their sizes (in bp) indicated. Arrows indicate the positions of polymerase chain reaction (PCR) primers. The male transcript is 739 bp, with vertical lines showing in‐frame stop codons in the male exon. The female transcript is 576 bp. (B) Sex‐specific splicing of *Dstra* was verified by reverse transcription (RT)‐PCR using different primer pairs (shown in A) to assess mRNA variants. Male‐specific products were obtained from wild‐type (WT) male complementary DNA (cDNA): *Dstra_F1/R1* = 419 bp, *Dstra_F1/R2* = 560 bp, *Dstra_F1/R3* = 715 bp. Female‐specific products were predominant in WT female cDNA: *Dstra_F1/R1* = 247 bp, *Dstra_F1/R2* = 388 bp, *Dstra_F1/R3* = 552 bp. Both male and female products were detected in mixed cDNA (male to female at 1:1 ratio). A unisex product (313 bp) was amplified using primer R3, with products less than 100 bp being primer dimers. (C) Sex‐specific *Dstra* products throughout development. Embryos were collected at different time points after egg laying (in hours), larvae (1st, 2nd, and 3rd instar), pupae (2 d after prepupae), adult females, and males. Primers for *Dstra* (*Dstra_F1/R3*), a male‐specific gene *β2‐tubulin* (*β2t*), a female‐specific gene *Yolk protein 1* (*Yp1*), and a housekeeping gene *TATA binding protein* (*TBP*) were used. PCR product sizes: 120 bp for *Dsβ2t*, 135 bp for *DsYp1*, and 182 bp for *TBP*. M is the molecular weight ladder.

The *Dstra* gene contains 3 protein‐coding exons, 2 introns, and 1 male‐specific exon (Fig. 1A). The male and female transcripts of *Dstra* are 739 bp and 576 bp, respectively, and the male exon contains several in‐frame stop codons. Sequence alignment of *tra* genes from selected *Drosophila* species revealed similar gene organization between *Dstra* and *Dmtra* (Fig. S1). The male‐ and female‐specific introns of *Dstra* are 68 and 231 bp long, respectively, slightly different from those of *Dmtra* (73 and 248 bp, respectively). The nonsex‐specific intron (intron 2) of *Dstra* is 381 bp, considerably larger than that of *Dmtra* (57 bp). The MEME analysis of male‐specific introns from 13 *Drosophila* species identified 3 motifs with a significant E‐value (Fig. S2). The 29 nt motif identified from all analyzed sequences contains the male‐specific splicing site, TTTTTGTTGTTTTTTTTCTAG, which is the binding site of the Sxl female‐specific protein, inhibiting male‐specific splicing of *tra* mRNA in females (Sosnowski *et al.*, 1989; Inoue *et al.*, 1990; Nagengast *et al.*, 2003).

In *D. melanogaster*, the male form of tra RNA can be detected from males, while both male and female forms of *tra* RNA were detected from females (Boggs *et al.*, 1987; Sosnowski *et al.*, 1989). When performing RT‐PCR with forward and reverse primers located on the 1st (Dstra_F1) and 2nd exon (Dstra_R1 or R2), a single product corresponding to male splicing was obtained from WT male cDNA. Meanwhile, a predominant female product along with the male product was obtained from WT female cDNA (Fig. 1B). Additionally, using primer Dstra_F1 and a reverse primer on the 3rd exon (Dstra_R3), a single male and female product was obtained from male and female cDNA, respectively. A unisex, nontarget amplification of 282 bp was also amplified when using the primer pair Dstra_F1 and Dstra_R3 (Figs. 1B and S3). The primer pair Dstra_F1 and Dstra_R3 was then used to assess the developmental expression profile of *Dstra* RNAs.

In several Dipteran species, such as housefly *Musca domestica* (Hediger *et al.*, 2010) and medfly *C. capitata* (Gabrieli *et al.*, 2010), the oriental fruit fly *B. dorsalis*, the guava fruit fly *B. correcta* (Liu *et al.*, 2015; Laohakieat *et al.*, 2020), and the common green bottle fly *Lucilia sericata* (Li *et al.*, 2013), maternal female form of *tra* RNA can be detected in the precellular embryos while the male form of *tra* RNA (coding nonfunctional product) showed up later at the onset of zygotic stage. In *D. melanogaster*, it is unclear if any *tra* RNA is maternally inherited (Verhulst *et al.*, 2010b). An analysis of transcriptome data throughout fly development showed that the *tra* transcript (nonsex‐specific) can be detected in early embryos (0–2 h), which reached moderately high levels in 4‐ to 10‐h‐old embryos and then decreased through embryogenesis (Graveley *et al.*, 2011), suggesting a “maternal female plus zygotic male” pattern of *tra* in *D. melanogaster*. In our study, the female transcript of *Dstra* was readily detectable in 0‐ to 1‐h‐old embryos (Fig. 1C), suggesting these were maternally inherited. Male transcripts were detected in 10‐ to 11‐h‐old embryos, reinforcing the “maternal female *tra* plus zygotic male *tra*” feature in *D. suzukii*. Female and male *Dstra* transcripts were present at lower levels during larval stages, and they accumulated to moderate levels at pupal and adult stages, mirroring the expression pattern of *Dmtra* at these stages (Graveley *et al.*, 2011). The expression of 2 sex‐specific genes, *β2‐tubulin* (*β2t*) and *Yolk protein 1* (*Yp1*), was detected starting from the 3rd instar larval stage and continuing into the pupal stage. Similar to *D. melanogaster*, *β2t* is expressed in adult males, while *Yp1* is expressed in adult females. These genes serve as controls for later developmental stages and different sexes.

### Transformer genes from Dipteran species are divergent

**Fig. 2:**
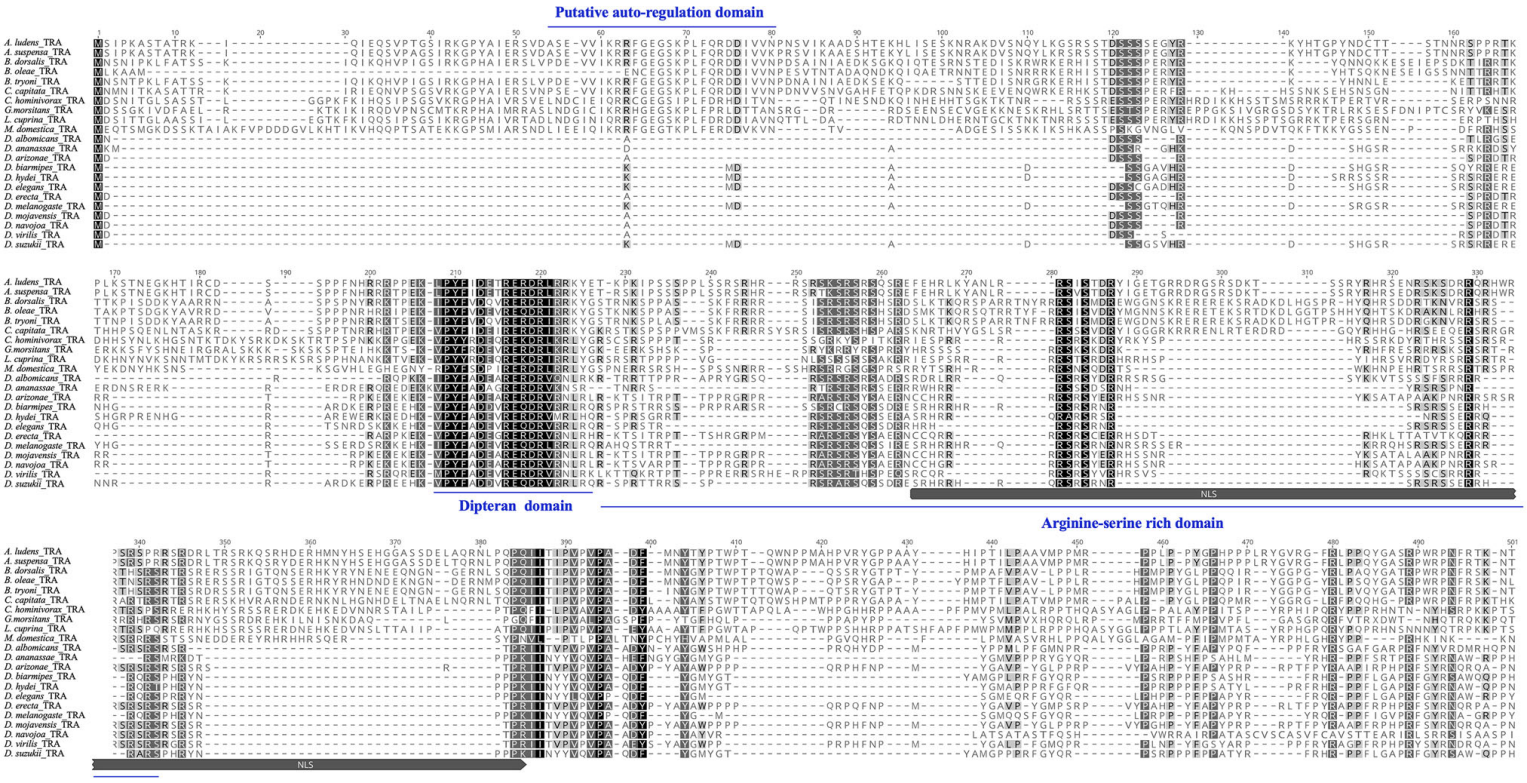
Alignment of TRANSFORMER (TRA) proteins from different Dipteran species. *Drosophila suzukii tra* protein sequence is aligned with orthologs from *D. albomicans* (XP_034109734.1), *D. ananassae* (XP_001957652.2), *D. arizonae* (XP_017860940.1), *D. biarmipes* (XP_016966065.1), *D. elegans* (XP_017128700.1), *D. erecta* (XP_001973038.2), *D. hydei* (XP_023165797.2), *D. melanogaster* (FBgn0003741), *D. mojavensis* (XP_002012186.1), *D. navojoa* (XP_017957514.1), *D. virilis* (XP_002046303.2), *Anastrepha ludens* (ABW04176.1), *A. suspensa* (AET31461.1), *Bactrocera dorsalis* (AKM21201.1), *B. tryoni* (XP_039965613.1), *B. oleae* (XP_036220685.1), *Ceratitis capitata* (XP_020714997.1), *Cochliomyia hominivorax* (AGE31793.1), *Glossina morsitans* (ACY40710.1), *Lucilia cuprina* (XP_023296766.1), and *Musca domestica* (ACY40709.1). The protein alignment was performed in Geneious Prime program using Geneious Alignment (Global). The conserved domains and the nuclear localization signal (NLS) are indicated.

The coding region sequence of the *Dstra* female form (*Dstra*‐CDS), resulting from alternative splicing, encodes a putative protein with 191 amino acids. This protein shows high similarity (87%) and identity (85%) to its ortholog in *D. biarmipes* (Figs. 2 and S4). Phylogenetic analysis of TRA proteins from 22 fly species revealed that all *Drosophila* TRA proteins form a distinct subgroup, while TRA proteins from Tephritid fruit flies, including *A. ludens*, *A. suspensa*, *B. dorsalis*, *B. tryoni*, *B. oleae*, and *C. capitata*, cluster into another subgroup (Fig. S5). Additionally, TRA proteins from *D. suzukii* and *D. biarmipes* clustered together (Fig. S5). Previously, low similarity (31%–36% identity) among TRA protein sequences from several *Drosophila* species was reported (Oneil & Belote 1992). The high degree of divergence, indicated by the overall low similarity (most similarity indices were less than 40%), was also observed among the analyzed TRA proteins (Fig. S4), highlighting the rapidly evolving nature of *tra*. Despite this, some conserved domains, including the putative autoregulation domain, the Dipteran domain, and the arginine‐serine‐rich domain, were identified through protein sequence alignment (Fig. 2). The autoregulation domains, spanning the 1st and 2nd exons, were found only in non‐drosophilid Diptera where *tra* is autoregulated. This domain may mediate complexes of TRA/TRA2/RNA binding protein 1 (RBP1) for the autoregulation of *tra* in females (Verhulst *et al.*, 2010a; Li *et al.*, 2013). The arginine‐serine‐rich domain is involved in targeting proteins to an RNA‐splicing‐related subnuclear compartment (Li and Bingham 1991). Indeed, the domain (SRHRRHRQRSRSRNRSRSRSSERRHRARSPHRYNPPPK) of the NLS from *Dstra* was successfully used to localize the protein into the cell nuclear in *D. suzukii* (Schwirz *et al.*, 2020). The Dipteran domains, present in all Dipteran TRA proteins, may provide the baseline activity of TRA. The observed high degree of divergence, alongside the presence of conserved domains in Dipteran TRA proteins, suggests that their roles in sexual development rely largely on functional domains rather than sequence structure.

### Ectopic expression of Dstra using Tet‐off system conditionally transformed the sex

Previously, plasmids expressing inverted repeats (IR) have been used to induce RNA interference (RNAi) of *tra* in *C. capitata* (Saccone *et al.*, 2007) and *L. cuprina* (Williamson *et al.*, 2021). In *L. cuprina*, a Tet‐off system was used to express *Lctra‐IR* conditionally, therefore transforming females into males (Williamson *et al.*, 2021). In *D. melanogaster*, introducing loss‐of‐function mutations into *tra* transformed females into males (Sturtevant, 1945; Belote *et al.*, 1990; Yuan & Belote, 1995; Carrami *et al.*, 2018; Kandul *et al.*, 2019), and the ectopic expression of *tra* transformed males into females (Mckeown *et al.*, 1988; An *et al.*, 2000; Savarit & Ferveur, 2002; Evans & Cline, 2007). Therefore, we built an IR including the whole CDS of *Dstra* (*Dstra‐CDS‐IR*) with the expectation that it will either produce double‐stranded RNA (dsRNA) or only CDS will be transcribed. Consequently, integration of *Dstra‐CDS‐IR* into the genome would lead to either silencing or ectopic expression of *Dstra*.

**Fig. 3:**
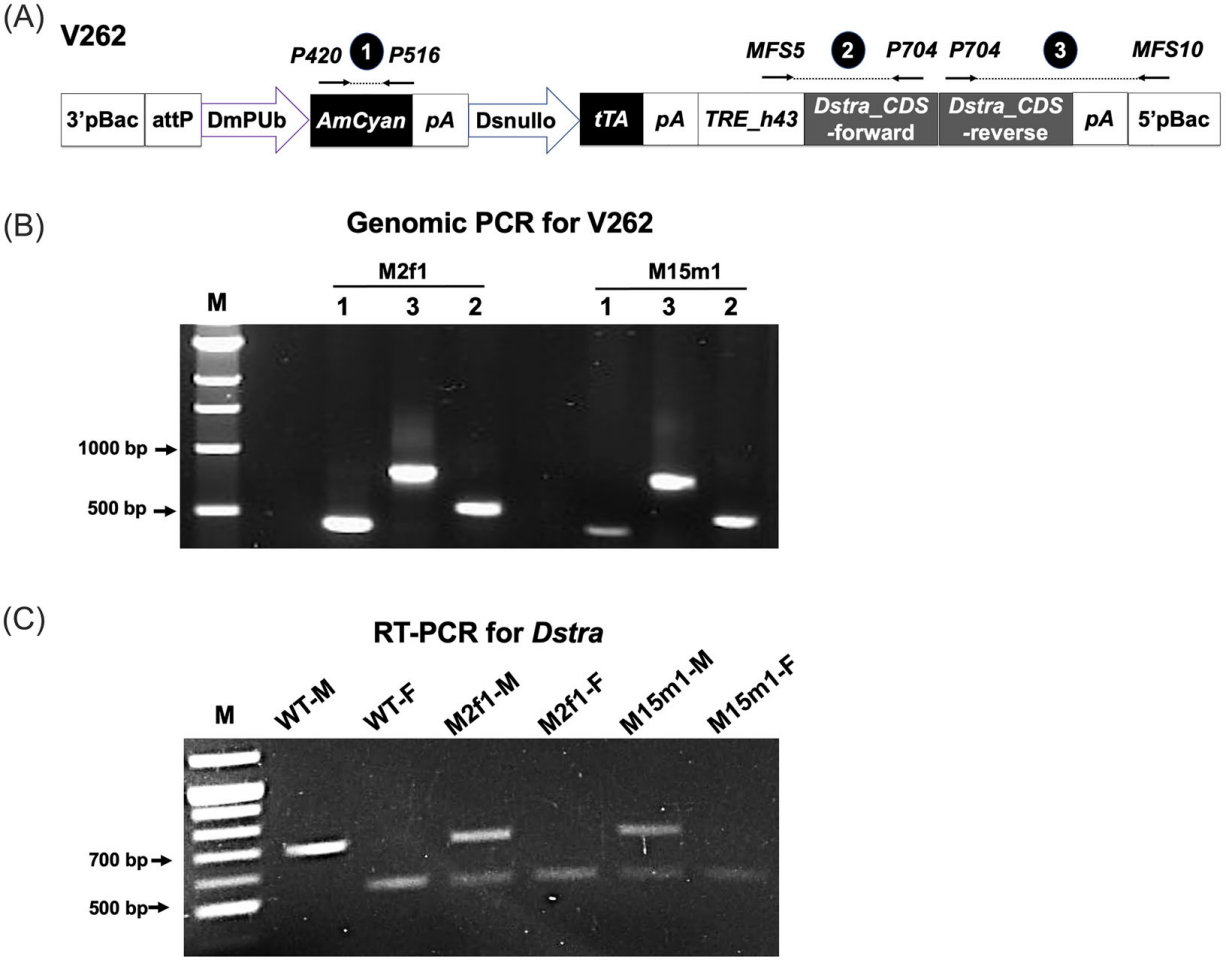
Molecular verification of transgenic *Drosophila suzukii* lines carrying inverted repeats of *Dstra* coding region sequence (CDS). (A) Schematic map of the piggyBac germ‐line transformation vector V262 (not to scale), containing an *AmCyan* marker gene regulated by the *D. melanogaster* polyubiquitin (PUb) promoter and an *attP* recombination site. V262 also includes a tetracycline transactivator (tTA) regulated by the *Dsnullo* promoter and an inverted repeat of the *Dstra* CDS fused to the tetracycline response element (TRE) with minimal *hsp43* promoter (TRE_hs43). Primer sets for genomic polymerase chain reaction (PCR) are indicated. (B) Genomic PCR confirms vector sequences in transgenic lines V262‐M2f1 and V262‐M15m1 using 3 primer sets: (1) P516/P420: 429 bp; (2) MFS5/P704: 533 bp; (3) P704/MFS10: 803 bp. (C) Detection of male (715 bp) and female (552 bp) *Dstra* transcripts in wild‐type (WT) and transgenic lines using primer set *Dstra_F1/R3*. M = molecular weight ladder.

**Fig. 4:**
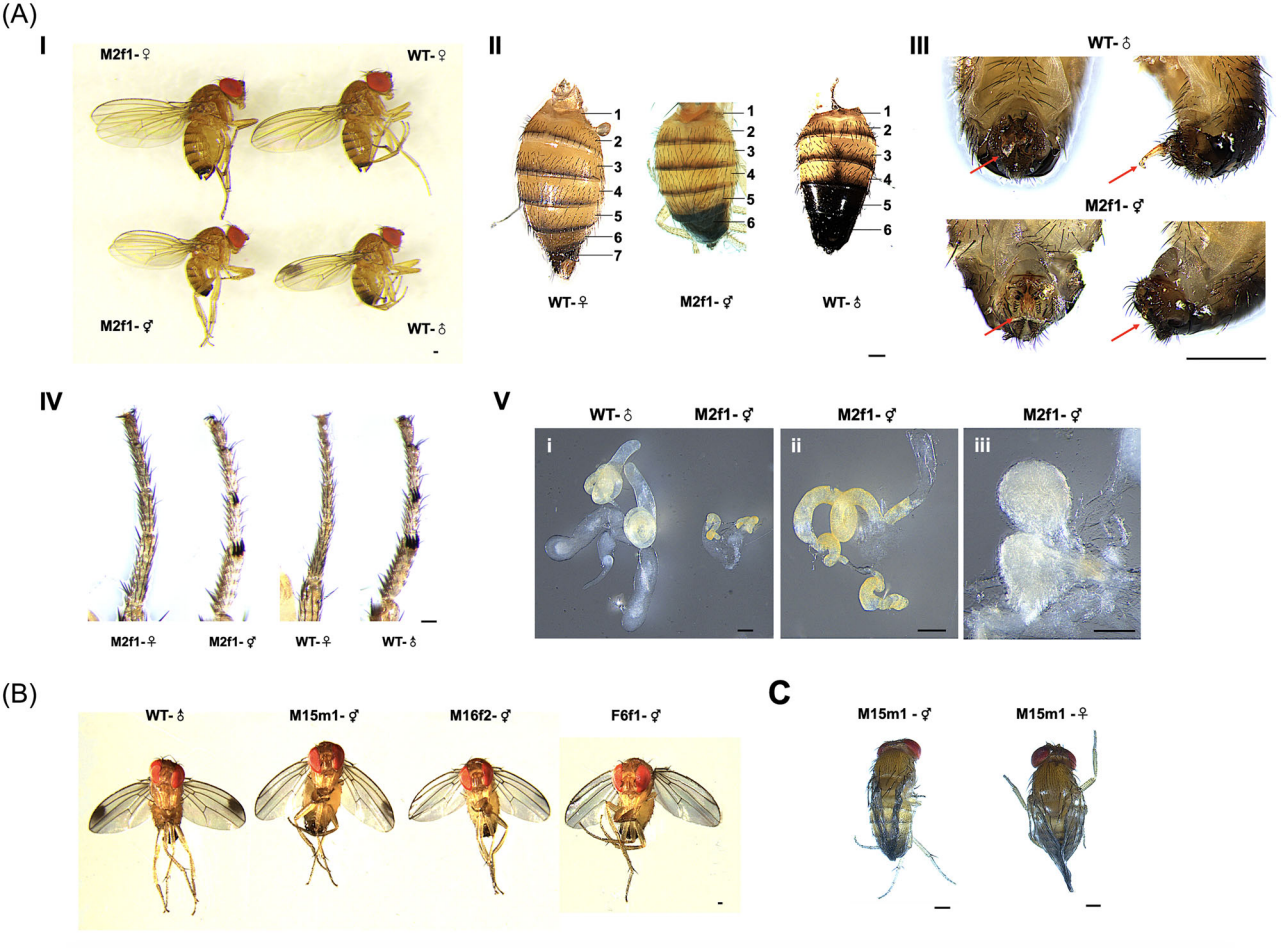
Transgenic *Drosophila suzukii* overexpressing *transformer* gene partially transforms the sex. (A) Morphological phenotypes for V262_M2f1 flies. I. V262_M2f1 males completely lost the black spots on the wings; II. V262_M2f1 males also developed feminized pigmentation indicated by the faint color from the 6th and 7th abdominal tergite segments; III. The aedeagus (indicated by red arrow) was always shown in wild‐type (WT) males but invisible in V262_M2f1 males; IV. V262_M2f1 males developed similar sex combs in the forelegs as in WT males; V. V262_M2f1 males developed either much smaller testis (i, right) compared to that from WT male (i, left), or asymmetric testis (ii), or ovaries (iii). (B) Male spotless phenotypes from transgenic lines V262_M15m1, V262_M16f2, and V262_F6f1. (C) Crippled wing phenotype for V262_M15m1 male and female. Scale bar: 0.2 mm.

We generated an all‐in‐one (AIO) Tet‐off vector, V262, where tTA is regulated by the cellularization gene promoter *Dsnullo* (Yan *et al.* 2020a) and *Dstra*‐CDS‐IR is fused to TRE (Fig. 3A). Four independent transgenic lines (M2f1, M15m1, M16f2, and F6f1) were created via germ‐line transformation using the V262 plasmid. Segregation analysis indicated that the transgene was inserted onto autosomes in each of the 4 lines, which were further bred to homozygosity using tetracycline‐supplemented food (100 *µ*g/mL). When tetracycline was omitted from the food in the parental generation, all male offspring from 3 lines (M16f2, M2f1, and F6f1) completely lost the spots on their wings, while female offspring exhibited a normal spotless phenotype (Fig. 4A, B), suggesting these males were partially transformed into pseudo‐females. This change in sexual dimorphism indicated that only the *Dstra* CDS (forward repeat of IR) was transcribed in these lines, leading to ectopic expression of *Dstra* in males. Interestingly, some male offspring from line M15m1 lost only 1 wing spot without tetracycline (Fig. 4B), and some male and female offspring exhibited abolished wing extension (Fig. 4C), suggesting a different ectopic expression profile of *Dstra* in M15m1 possibly due to chromosomal position effects. Further analysis, such as inverse PCR, could be performed to identify the genomic integration site of the transgene (Yan & Schetelig, 2024). This may help to clarify further how the transgene insertion site influenced the morphological phenotypes in V262.

Lines M2f1 and M15m1 were chosen for molecular verification of the *Dstra* IR cassette transcription. Genomic PCRs with internal primer pairs producing 3 amplicons spanning the marker gene *AmCyan* and *Dstra* IR cassettes were performed. PCR products (Fig. 3B) were sequenced and confirmed the integrity of these cassettes. RT‐PCR using primers *Dstra_F1* and *Dstra_R3* showed that while male and female *Dstra* transcripts were detected in WT males and females, respectively, both transcripts were detected in transgenic males (Fig. 3C), confirming the production of female *Dstra* RNA in these males (hereafter referred to as *Dstra*+ males). Anatomical examination identified female morphology in *Dstra*+ males (Fig. 4A). For instance, all M2f1 males developed feminized pigmentation, indicated by the faint color from the 6th and 7th abdominal tergite segments. After a cold shock (−20 °C for 2 min), all WT males exhibited their aedeagus, while none of the M2f1 males did, suggesting abnormal external genitalia structure. These pseudo‐females developed either much smaller or asymmetric testes, or premature ovaries.

**Fig. 5:**
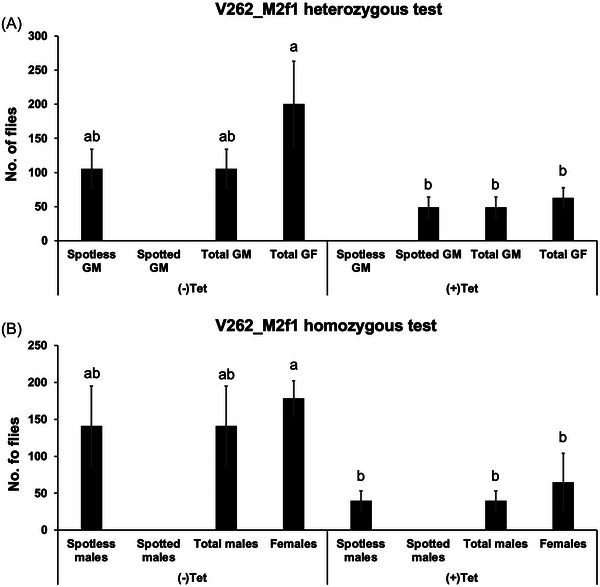
Genotype and phenotype verification for V262_M2f1 flies on diet with (+Tet) or without (–Tet) tetracycline. For the heterozygous test (A), 5 V84_F15f1 males (X‐linked, carrying *DsRed* marker) were crossed with 10 V262_M2f1 virgin females. The number of genetic males (GM, no red fluorescence) and females (GF, red fluorescence) were counted. The number of genetic males with (spotted) or without (spotless) black spots on the wings was verified. For the homozygous test (B), 5 homozygous males were crossed with 10 homozygous females. The number of spotless males, spotted males (2 spots), and females were counted. The mean and standard error from 3 replicate experiments are shown. Different lower‐case letters indicate significant differences (*P* < 0.05, one‐way analysis of variance).

To verify if these pseudo‐females were derived from genetic males, males from an X‐linked line V84_F15f1, which carries a DsRed marker, were crossed with virgin females from the homozygous V262_M2f1 line. Consequently, in the next generation, genetic females would carry 1 copy of each V84 and V262 transgene, while genetic males would carry only 1 copy of the V262 transgene. Without tetracycline, all genetic males (showing no red fluorescence) developed into spotless pseudo‐females, while a similar number of genetic females (showing red fluorescence) were also produced (one‐way ANOVA, *P* = 0.098) (Fig. 5A). With tetracycline, all genetic males developed a normal (spotted) wing phenotype, indicating the ectopic expression of *Dstra* was switched off when flies carried 1 copy of the V262 transgene. The number of genetic males (*P* = 0.319) and females (*P* = 0.032) from the Tet‐diet were lower than those from the tetracycline‐free diet in the heterozygous condition (Fig. 5A). For homozygous V262_M2f1 flies, a similar number of spotless males and females were produced without tetracycline (*P* = 0.543). However, placing V262_M2f1 flies on Tet‐diet still produced spotless males, suggesting leaky expression of *Dstra* in homozygotes that cannot be fully suppressed by tetracycline. Similarly, the total number of males (*P* = 0.060) and females (*P* = 0.039) from Tet food was lower than those from the tetracycline‐free diet in the homozygous condition (Fig. 5B), indicating some deleterious effects of Tet on V262_M2f1 flies.

### Ectopic expression of Dstra affected the splicing of Dsfru and mating behavior in males

**Fig. 6:**
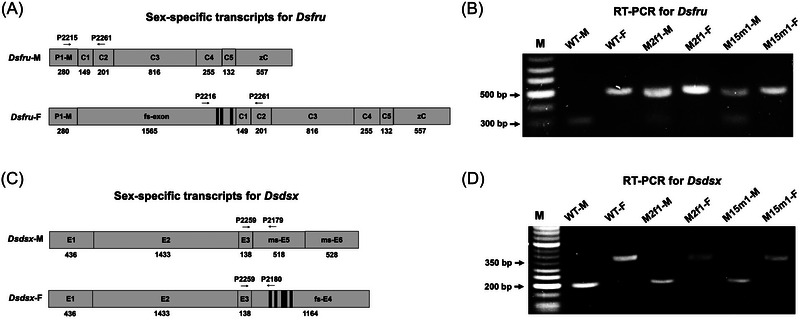
Sex‐specific transcripts of *Dsfru* and *Dsdsx* genes in the wild‐type (WT) and *Dstra^+^* lines. (A) Due to the large size and complex structure of the *fru* gene, only partial and conserved structures of different messenger RNA (mRNA) variants are shown. *Dsfru* male transcript contains 7 exons, including exon P1‐M next to the most distal *fru* promoter (P1), 5 common exons (C1–C5), and a terminal exon zC encoding the type C zinc‐finger domain. The female transcript contains a female‐specific (fs) exon, all common exons, and the zC exon. Exon sizes are predicted based on *fru* mRNA variants from *Drosophila suzukii* (LOC108020297) and *D. melanogaster* (FBgn0004652). Reverse transcription – polymerase chain reaction (RT‐PCR) primer positions are indicated. (B) RT‐PCR detection of *Dsfru* transcripts from WT and transgenic lines V262‐M2f1 and V262‐M15m1. Primer set P2215/P2261 (320 bp) is used for GM (M), and P2216/P2261 (558 bp) for genetic females (F). (C) *Dsdsx* male transcript contains 3 common exons followed by male‐specific exons (E5 and E6), while the female transcript contains 3 common exons followed by a female‐specific exon (E4). Exon sizes are predicted based on *dsx* mRNA variants from *D. suzukii* (LOC118876773 and LOC108011781) and *D. melanogaster* (FBgn0000504). RT‐PCR primer positions are indicated. (D) RT‐PCR detection of *Dsdsx* transcripts from WT and transgenic lines. Primer set P2259/P2179 (204 bp) is used for GM (M), and P2259/P2180 (376 bp) for genetic females (F). M = molecular weight ladder. Exons are drawn to scale with sizes indicated. Black bars within exons indicate the 13 nt TRANSFORMER (TRA)/TRA2 binding site: A/T‐C‐A/T‐A/T‐CAATCAACA.

In *D. melanogaster*, *tra* interacts with *tra2* to regulate the sex‐specific splicing of 2 downstream genes, *fru* and *dsx*. The TRA/TRA‐2 complex binds to 13 nt cis‐acting elements present only in the female‐specific exons of *fru* and *dsx*, thereby activating the female‐specific splicing of these pre‐mRNAs (Hoshijima *et al.*, 1991; Ryner & Baker, 1991; Heinrichs *et al.*, 1998; Gailey *et al.*, 2006). Three and 5 TRA/TRA‐2 binding sites were identified in the female‐specific exons of *Dsfru* and *Dsdsx*, respectively (Fig. 6A, C). Using forward primers specific to the male (*P1‐M*) and female‐specific exon of *Dsfru*, and a reverse primer on the common exon C2, male (*DsfruM*) and female (*DsfruF*) transcripts were amplified from WT males and females, respectively (Fig. 6B). Interestingly, both *DsfruM* and *DsfruF* were also obtained from *Dstra*+ males, indicating that female‐specific splicing of *Dsfru* was initiated in these male flies. For *Dsdsx*, a forward primer on the common exon E3 and reverse primers specific to the male (E5) and female (E4) exons were used to detect sex‐specific transcripts from WT flies (Fig. 6C). Unlike *Dsfru*, where both male and female transcripts were amplified from *Dstra*+ males, only male *Dsdsx* products were detected (Fig. 6D), suggesting that the ectopic expression of *Dstra* did not affect the splicing of *Dsdsx* in these flies.

**Fig. 7:**
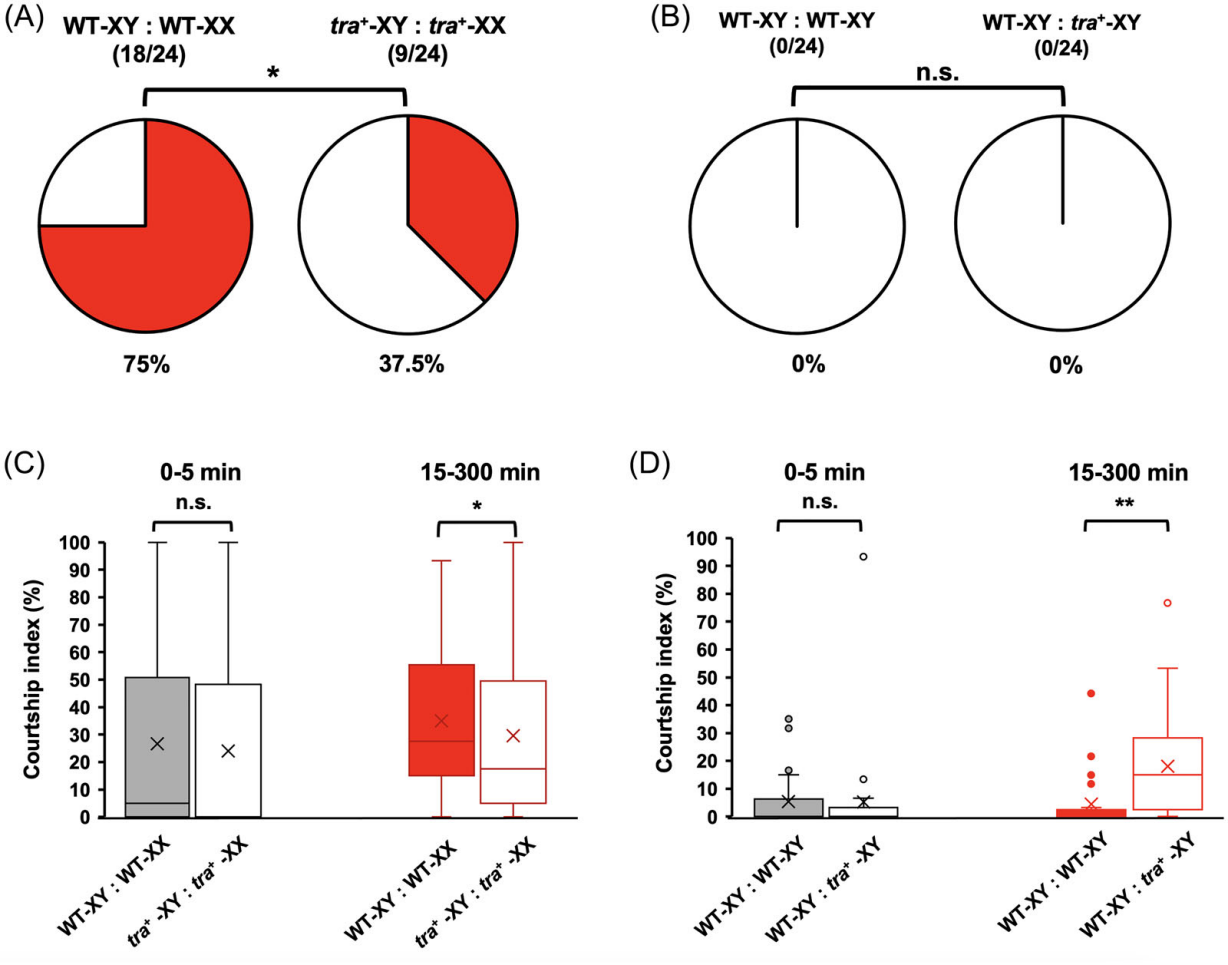
Sexual behavior analysis. Copulation success was recorded for pairs of genetic males and females (A), as well as for pairs of genetic males (B). In the same experiments, Courtship index (CI) within the 1st 5 min and 15–300 min period was scored (C and D). The genotypes of the flies used for analysis are indicated. For each group, *n* = 24 were analyzed. **P < 0.001; *P < 0.05; n.s. = not significant (Chi‐square test).

In *D. melanogaster*, *fru* is considered the “switch gene” for determining sexually dimorphic behaviors through the central nervous system (CNS) (Billeter *et al.*, 2006; Yamamoto, 2008; Baker *et al.*, 2024). The sex‐specific splicing of *fru* specifies its transcripts in the brain and the male CB (Ryner *et al.*, 1996; Demir and Dickson 2005). Loss‐of‐function alleles of the *fru* gene disrupt male CB and sexual orientation. Ectopic *fru* expression in females results in male‐like characteristics, such as the formation of the male‐specific muscle of Lawrence (MOL) (Usui‐Aoki *et al.*, 2000; Demir & Dickson, 2005; Billeter *et al.*, 2006). Since overexpression of *Dstra* altered the splicing pattern of *Dsfru* in males (Fig. 6B), we examined its effects on the sexual behavior of these flies. In small arenas, copulation was observed in 14 out of 24 WT pairs (58.3%) within 60 min, while it occurred in only 2 out of 24 *Dstra*+ pairs (8.3%) (Fig. S6A). Within 300 min, 75% of WT pairs and 37.5% of *Dstra*+ pairs copulated (Fig. 7A). *Dstra*+ males showed similar levels of courtship initiation (CI) (26.7%) compared to WT males (24.0%) within 5 min (Fig. 7C), although most of these CB did not lead to copulation within 60 min (Fig. S6A). Between 15 and 300 min, *Dstra*+ males exhibited significantly lower CI (29.5%) compared to WT males (34.9%) (Fig. 7C). When 2 genetic males were housed together, no copulation was observed (Figs. S6B and 7B). Within 5 min, WT males showed similarly low levels of CI (5.3%) when paired with either another WT or *Dstra*+ male. Interestingly, over a longer period (15–300 min), the CI of WT males paired with *Dstra*+ males increased to 18%, significantly higher than the 4.4% observed when WT males were paired with other WT males. This indicates that WT males showed more interest in courting *Dstra*+ males. These results suggest that the sexual behavior and orientation (attracting males rather than females) of *Dstra*+ males were disrupted.

## Discussion

In *D. melanogaster*, a double dose of X‐chromosome signal elements (XSEs) activates the sexual pathway establishment promoter, *SxlPe*, only in diploid XX individuals. Conversely, a single dose of XSEs is insufficient to activate *SxlPe*, resulting in no *Sxl* product in the early embryo of haplo‐X individuals. Following initial expression controlled by *SxlPe*, *Sxl* is regulated by the sexual pathway maintenance promoter, *SxlPm*, in both males and females. Notably, *Sxl* undergoes sex‐specific splicing, producing a truncated product in males and a functional product in females (Cline & Meyer, 1996; Penalva & Sanchez, 2003; Pomiankowski *et al.*, 2004). In addition, *Sxl* is autoregulated, with its functional product (Sxl‐F) present throughout female development, acting on the female‐specific pre‐mRNA splicing of the downstream gene *tra*. In many Dipteran species where *tra‐F* instead of *Sxl‐F* is autoregulated, maternal *tra* mRNA or protein is detected and considered the initiating signal for its autoregulation (Verhulst *et al.*, 2010a; Bopp *et al.*, 2014). However, it is unclear if TRA‐F is maternally passed to the embryo in *D. melanogaster* (Belote *et al.*, 1989; Siera & Cline 2008; Verhulst *et al.*, 2010a). Here, we detected female, but not male, *Dstra* RNA in the prezygotic stage (Fig. 1C), suggesting that this RNA was maternally deposited in *D. suzukii*.

While it is generally believed that the regulation pathway flows one way from *Sxl* to *tra*, a genetic study showed the reciprocal regulatory effect of *tra* on *Sxl* in *D. melanogaster* (Siera & Cline, 2008). Specifically, the ectopic expression of *tra‐F* positively stimulated the autoregulation of *Sxl‐F*, possibly by binding to the conserved TRA‐F binding sites, TC(T/A)(T/A)C(A/G)ATCAACA, located in the male‐specific exon of *Sxl*. Binding of TRA‐F to these sites may inhibit splicing to the male exon, thus promoting the splicing of *Sxl‐F*. Consequently, transgenic maternal or zygotic TRA‐F rescued the ovaries of *Sxl*‐deficient mutants by affecting *Sxl* autoregulation in the gonadal soma (Siera & Cline, 2008). It is possible that TRA‐F also influences *Sxl‐F* autoregulation in *D. suzukii*, which can be verified by the ectopic expression of TRA‐F in an *Sxl*‐deficient genetic background of female individuals. For this purpose, the V262 strains obtained in this study can be used as a transgenic source of TRA‐F, while viable strains carrying *Sxl* mutations have yet to be generated.

Previously, transgenic *D. melanogaster* lines expressing *hsp70‐tra‐F* were generated, which induced female development in WT males and also rescued the sex reversal phenotype in *tra^−^* flies (Mckeown *et al.*, 1988; Belote *et al.*, 1989). Additionally, the ectopic expression of *tra‐F* using the UAS‐Gal4 system is both sufficient and necessary to generate bisexual behavior in *D. melanogaster* (An *et al.*, 2000; Savarit & Ferveur, 2002). The morphological and behavioral phenotypes of the pseudo‐females from V262 strains suggest that *Dstra* controls the development of sexually dimorphic tissues and copulation behaviors in *D. suzukii*. Similar intersex phenotypes were observed when a temperature‐sensitive point mutation was introduced into *tra2*, the partner gene of *tra*. Heat shock treatments (26 to 29 °C) of *D. suzukii* carrying this mutation previously produced sterile pseudo‐females (Li & Handler, 2017). An earlier study generated haplo‐X/diplo‐X mosaic *D. melanogaster* flies, and expressing *tra* in the somatic cells (from haplo‐X male) is sufficient to elicit a fully feminizing nonautonomous signal for germ cells (from diplo‐X female) to make functional eggs (Evans & Cline, 2007). The observation that some V262 pseudo‐females developed ovaries (Fig. 4D) suggests that *Dstra* was expressed in somatic and/or germ‐line cells, promoting oogenesis in these chromosomal males.

An intriguing finding of this study is that the ectopic expression of *Dstra* affected the sex‐specific splicing of *Dsfru* but not *Dsdsx* in adult males (Fig. 6B, D). It is believed that in *Drosophila*, male sexual behavior is predominantly determined by *fru* through the CNS, whereas *dsx* regulates somatic sexual differentiation in both sexes outside the CNS (Burtis & Baker, 1989; Ryner *et al.*, 1996; Baker *et al.*, 2001; Demir & Dickson, 2005; Salvemini *et al.*, 2010; Peng *et al.*, 2021; Baker *et al.*, 2024). It is possible that the female product of *Dsdsx* (*Dsdsx‐F*) was produced at preadult stages, leading to some female anatomy in *Dstra*+ males. The absence of *Dsdsx‐F* in adults may explain why *Dstra*+ males are only partially transformed into females (Fig. 4). On the other hand, both *Dsfru‐F* and *Dsfru‐M* were obtained from *Dstra*+ males. In *D. melanogaster*, *fru‐F* is not translated into a functional protein, as only mRNA, not protein, from *fru* is detected in the female CNS (Usui‐Aoki *et al.*, 2000; Demir & Dickson, 2005). This was also confirmed in *D. virilis*, and the male‐specific expression of the Fruitless protein seems to be conserved in *Drosophila* species (Usui‐Aoki *et al.*, 2000; Billeter *et al.*, 2006; Gailey *et al.*, 2006; Baker *et al.*, 2024). A systematic study investigated sex‐ and tissue‐specific FRU expression in 7 *Drosophila* species. Surprisingly, only *D. suzukii* showed FRU expression in the female brain, lamina, and ventral nerve cord. No FRU expression was detected in the other 5 species, while *D. yakuba* exhibited some expression only in the retina (Yamamoto *et al.*, 2004). Therefore, it is likely that *Dsfru‐F* produces a functional protein in the CNS that regulates mating in *D. suzukii* females, and the expression of *Dsfru‐F* in *Dstra*+ males may explain the interruption of their sexual behaviors.

An interesting phenotype in these *Dstra*+ males is the loss of spots on their wings (Fig. 4). *D. suzukii* and *D. biarmipes* are closely related, and both show black spots on male wings. This phenotype is partially controlled by the pigmentation gene *yellow* (*y*), as *y^−^* mutants of *D. suzukii* and *D. biarmipes* exhibit faint coloration of these spots (Hinaux *et al.*, 2018; Yan *et al.*, 2021a). Another study verified that *y* expression directly controls the spotted‐wing pattern in *D. guttifera*, which can be sufficiently induced by ectopic expression of the *wingless* gene (Werner *et al.*, 2010). It has also been reported that *y* is a downstream gene of *fru* and plays a role in the mating success in *D. melanogaster* (Drapeau *et al.*, 2003; Massey *et al.*, 2019). Specifically, *y* is upregulated by FRU‐M in the CNS of WT males, whereas certain *fru* mutants completely abolished wing extension during courtship. In these mutants, *y* was not upregulated, but ectopic expression of *y* rescued male wing extension (Drapeau *et al.*, 2003). Since the sex‐specific splicing of *Dsfru* was interrupted in *Dstra*+ males (Fig. 6), it is possible that *y* was mis‐regulated in these flies, leading to the spotless phenotype. Meanwhile, the sex determination genes, especially *dsx*, were found to control the sex‐specific growth of the genital disc via Wingless and Decapentaplegic signaling at early stages in *D. melanogaster* (Keisman & Baker, 2001; Sánchez *et al.*, 2001). Therefore, the ectopic expression of *Dstra* in M15m1 flies may regulate downstream genes such as *dsx*, *y*, and *wingless* differently, causing the single spotted‐wing or deformed‐wing phenotypes (Fig. 4B, C). Collectively, our data strongly suggest a functional link between *tra*, *fru*, *dsx*, *y*, wing extension, pigmentation, and sexual behavior in *D. suzukii*.

The isolation and functional verification of the *Dstra* gene have significant implications for pest control in *D. suzukii* (Yan *et al.*, 2024). The *Dstra* gene plays a crucial role in sexual development, and understanding its mechanisms opens up several avenues for genetic control strategies. First, the female‐specific intron of the *Dstra* gene can be utilized in genetic control methods such as fs‐RIDL and TESS. Previous studies have successfully used the *traF* intron from *C. capitata* and *C. hominivorax* in *D. suzukii* for engineered female‐specific lethality (Li *et al.*, 2021; Schetelig *et al.*, 2021). Although the *D. suzukii traF* intron did not mediate female‐specific expression of *hid^Ala4^* in initial attempts, further optimization may enhance its effectiveness (Schetelig *et al.*, 2021). Second, the C‐terminal NLS from *Dstra* has shown efficacy in directing proteins to the cell nucleus (Schwirz *et al.*, 2020). This element can be fused to Cas9 to improve genome editing efficiency (Cong *et al.*, 2013; Hu *et al.*, 2018), potentially increasing the effectiveness of CRISPR‐based control strategies (Yan *et al.*, 2023). Third, an SRD strategy could be developed by targeting *Dstra*, for example, using a transgenic RNAi approach. A transgenic RNAi system employing a Tet‐Off mechanism has been demonstrated in *L. cuprina*. In this system, the constitutive promoter *Lsspt* outperformed the embryonic promoter *Lsbnk*, possibly due to its activity in the female germ‐line, which may contribute to a maternal effect (Williamson *et al.*, 2021). In this context, the *Dsnullo* promoter could be a suitable candidate for controlling the IR or hairpin targeting *Dstra* via a Tet‐Off system due to its constitutive activity. Fourth, introducing mutations in *Dstra* can result in sex reversal and sterility. For example, pgSIT targeting *tra* in *D. suzukii* successfully transformed and sterilized females (Kandul *et al.*, 2022). A recent study targeting *dsxF* with a split gene drive resulted in female sterility in *D. suzukii*. Modeling indicated that this strategy could effectively achieve population suppression (Yadav *et al.*, 2023).

In summary, this study elucidated the gene structure, splicing pattern, and biological functions of *Dstra* in sexual development. DNA elements of *Dstra*, such as the *traF* intron and NLS, provide valuable tools for genetic engineering and genome editing in *Drosophila* and potentially other Dipteran species. The V262 lines generated here offer genetic tools to investigate critical biological processes, including sexual dimorphism, copulation behaviors, wing extension, and pigmentation. Genetic control strategies targeting *Dstra* show significant promise for species‐specific and sustainable pest management.

### GenBank accession numbers

The GenBank accession numbers are as follows: *Dstra* male mRNA: MN982936.1; *Dstra* female mRNA: MN982935.1; *Dstra* gene: MN982934.1; V262: MN735455.

## Author contributions

Y.Y., J.Z., and J.S. performed the molecular works. Y.Y., J.Z., and C.B. carried out biological experiments. Y.Y., C.L., and B.L. conducted bioinformatic analysis. Y.Y. conceived of the study and analyzed data. Y.Y., W.Q., F.W., and M.F.S. coordinated the project and wrote the manuscript. All authors read and approved the final manuscript.

## Disclosure

The authors declare they have no conflict of interest.

## Supporting information




**Fig. S1** Organization and comparisons of *transformer* (*tra*) genes from different *Drosophila* species. Gene sequences were downloaded from the National Center for Biotechnology Information or Flybase (species names and sequence ID are shown in the figure) and aligned to the *D. suzukii tra* gene sequence obtained in this study using Clustal Omega. The exons and introns from *D. melanogaster* and *D. suzukii tra* genes are indicated.

**Fig. S2** MEME analysis of male‐specific introns from *transformer* (*tra*) genes. (A) Multiple sequence alignments of *tra* male introns. Identical amino acids are highlighted in black, and similar amino acids are shown in gray. (B) Conserved motifs in the male exons and introns identified by MEME analysis. Three motifs with a E‐value of significance were identified. The motif (29 nt) with the lowest E‐value (2.3e‐079) was identified from all 13 analyzed sequences, the remaining 2 motifs (29 nt and 18 nt for the length, 8.1e‐014 and 2.7e‐002 for the E‐value, respectively) were identified from 5 analyzed sequences.

**Fig. S3** Verification of the unisex polymerase chain reaction (PCR) product from the reverse‐transcript (RT)‐PCR against *Dstra* gene. (A) Alignment of the *Dstra* male transcript, female transcript, and the unisex PCR product (Multiple Alignment using Geneious software). Low similarities were identified between the unisex product and the *Dstra* male (27%) or female (34%) transcript. (B) Alignment of the unisex PCR product and corresponding primer sequences (Map to Reference using Geneious software), indicating that this unisex product is a nonspecific amplification of the corresponding primers.

**Fig. S4** Distance matrix of TRANSFORMER (TRA) proteins from different Dipteran species. See Fig. 2 legend for the sequence identifiers and alignment methods.

**Fig. S5** Phylogenetic analysis of dipteran TRANSFORMER (TRA) proteins. Unrooted neighbor‐joining tree was constructed using TRA amino acid sequences. Bootstrap values (1 000 replicates) are shown on the nodes of the trees. See Fig. 2 legend for the sequence identifiers and alignment methods.

**Fig. S6** Analysis for copulation success. Twenty‐four pairs of genetic male and female (A) and 24 pairs of genetic males (B) were recorded for 300 min. The red bar indicates the copulation period, whereas the gray bar indicates no copulation. The genotypes of the flies used for analysis are indicated.

**Table S1** Primer sequences
