# Supplementary figures and images for: The transformer gene controls sexual development in Drosophila suzukii
Source: Insect Sci. 2025 Mar 30;33(2):547–65. doi: 10.1111/1744-7917.70031 (PMC13087859; doi:10.1111/1744-7917.70031)

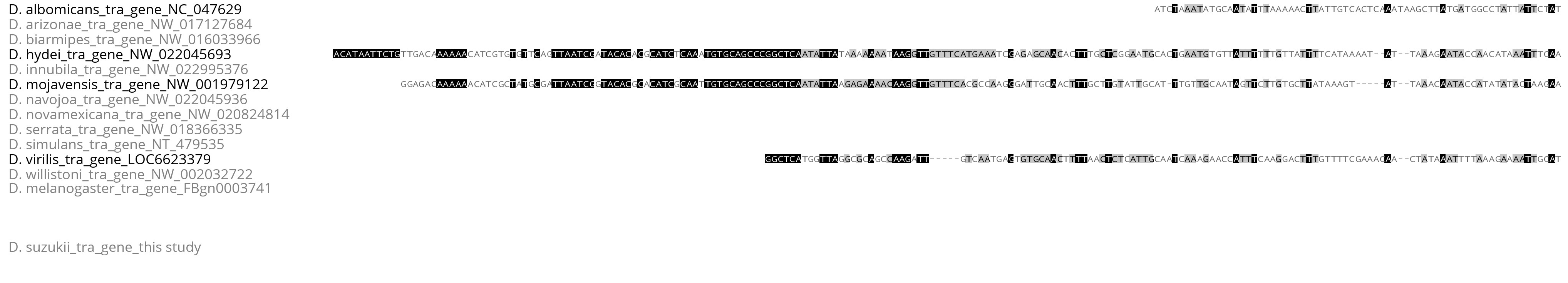


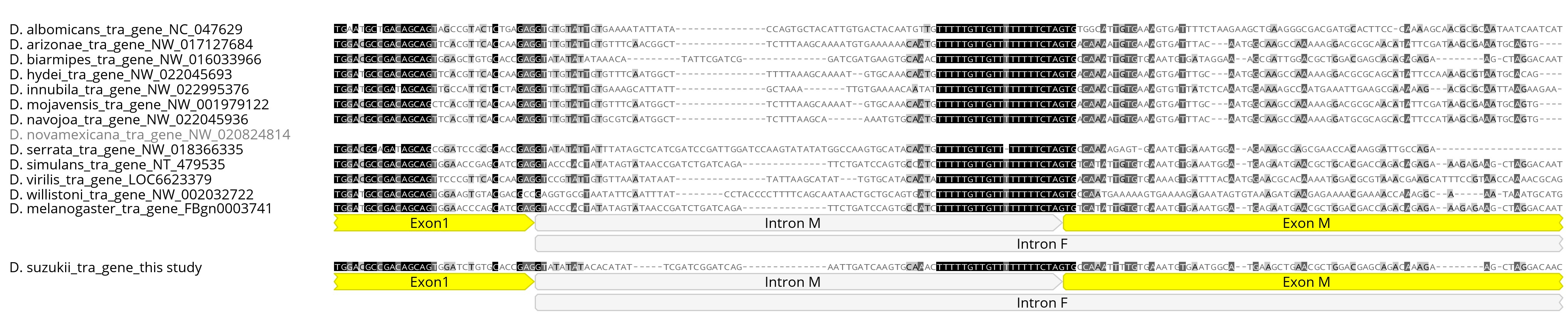


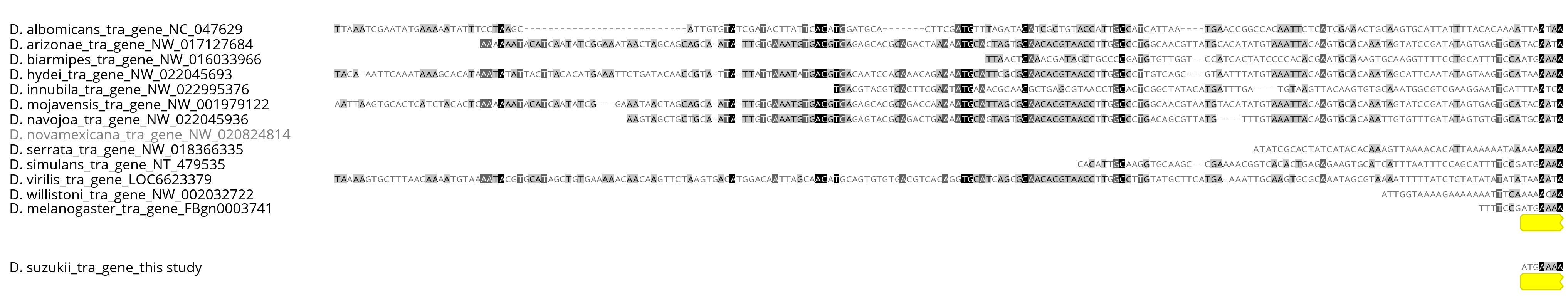


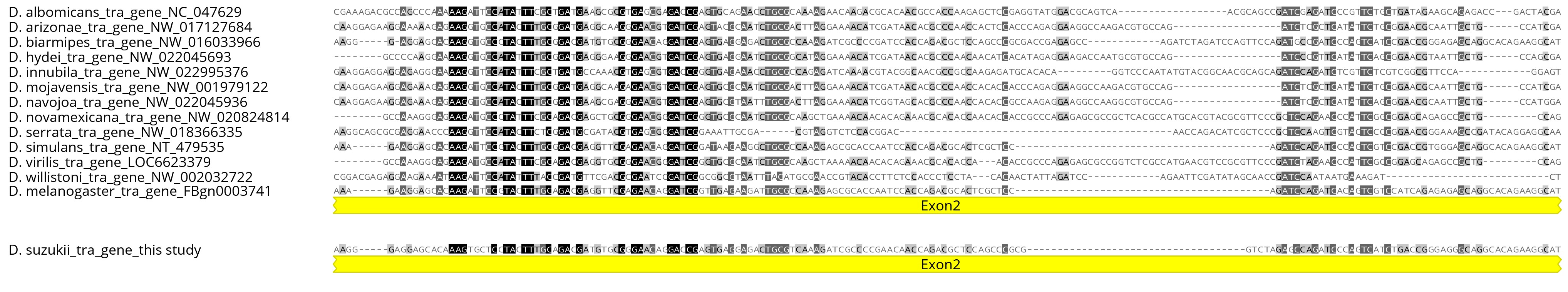


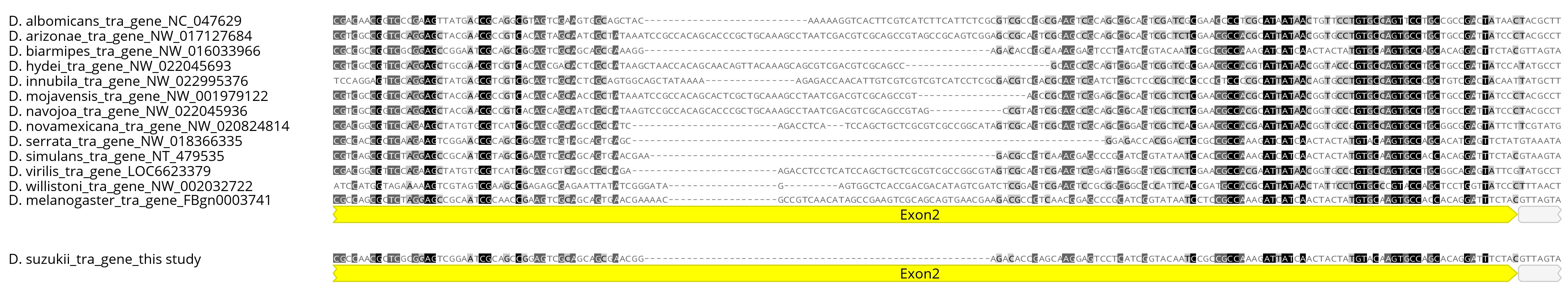


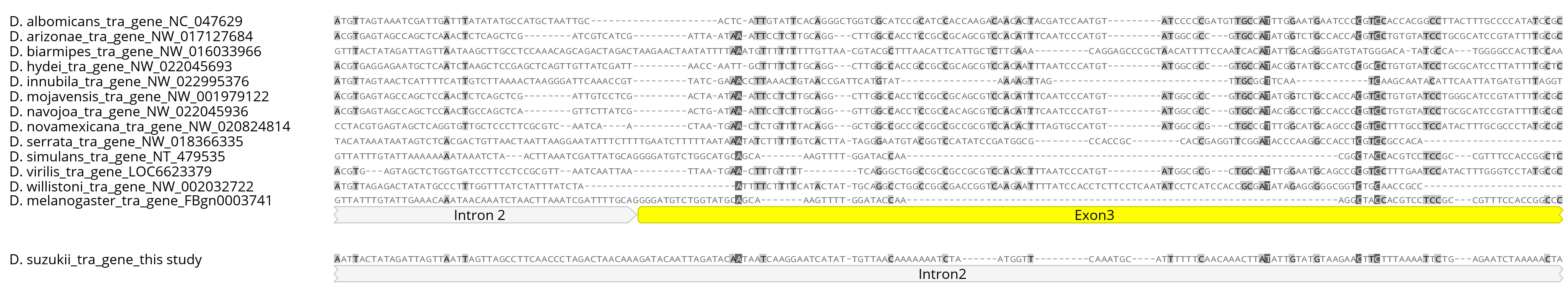


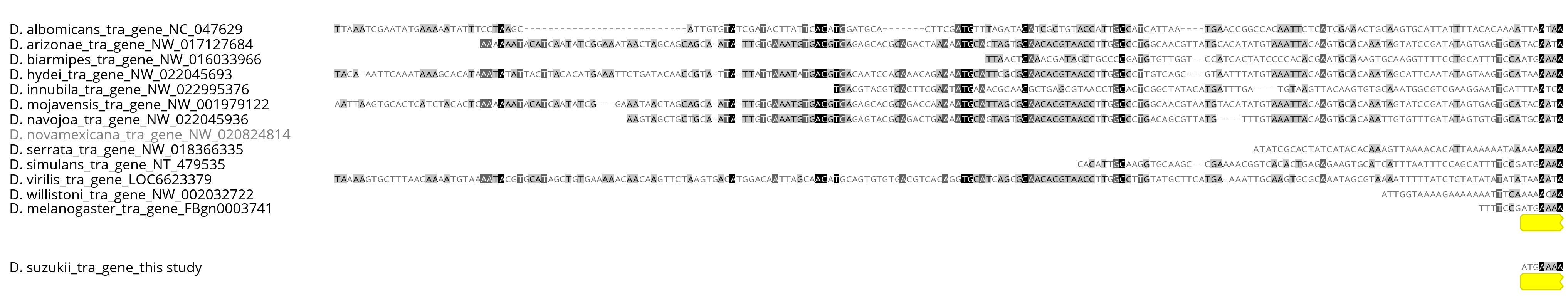


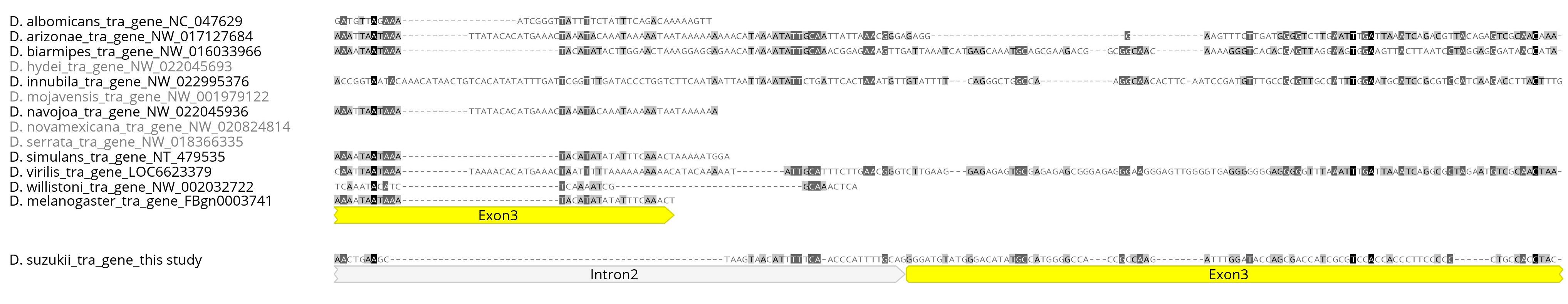


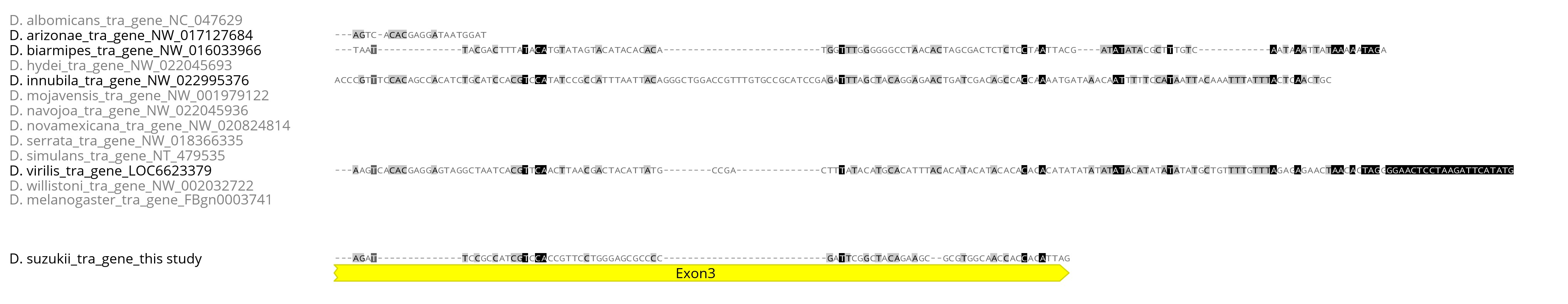

Supplement: Supplementary file 1 — Fig. S1 Organization and comparisons of transformer (tra) genes from different Drosophila species. Gene sequences were downloaded from the National Center for Biotechnology Information or Flybase (species names and sequence ID are shown in the figure) and aligned to the D. suzukii tra gene sequence obtained in this study using Clustal Omega. The exons and introns from D. melanogaster and D. suzukii tra genes are indicated. [file INS-33-547-s007.docx]

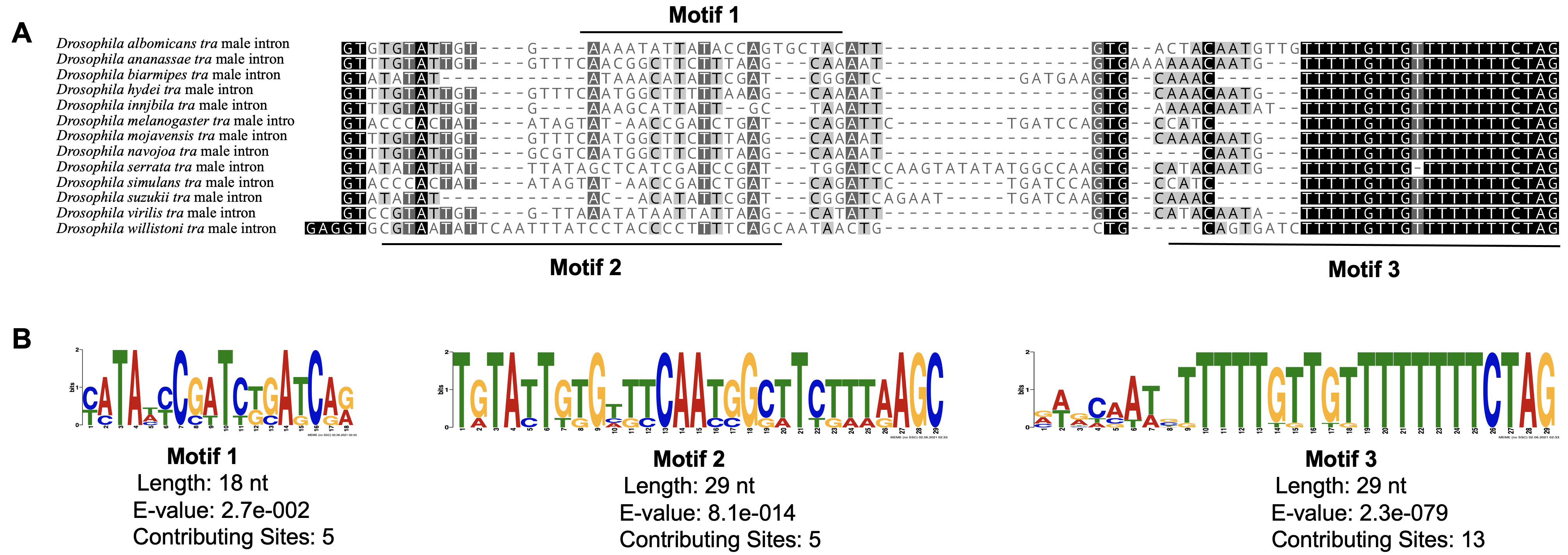

Supplement: Supplementary file 2 — Fig. S2 MEME analysis of male‐specific introns from transformer (tra) genes. (A) Multiple sequence alignments of tra male introns. Identical amino acids are highlighted in black, and similar amino acids are shown in gray. (B) Conserved motifs in the male exons and introns identified by MEME analysis. Three motifs with a E‐value of significance were identified. The motif (29 nt) with the lowest E‐value (2.3e‐079) was identified from all 13 analyzed sequences, the remaining 2 motifs (29 nt and 18 nt for the length, 8.1e‐014 and 2.7e‐002 for the E‐value, respectively) were identified from 5 analyzed sequences. [file INS-33-547-s002.jpg]

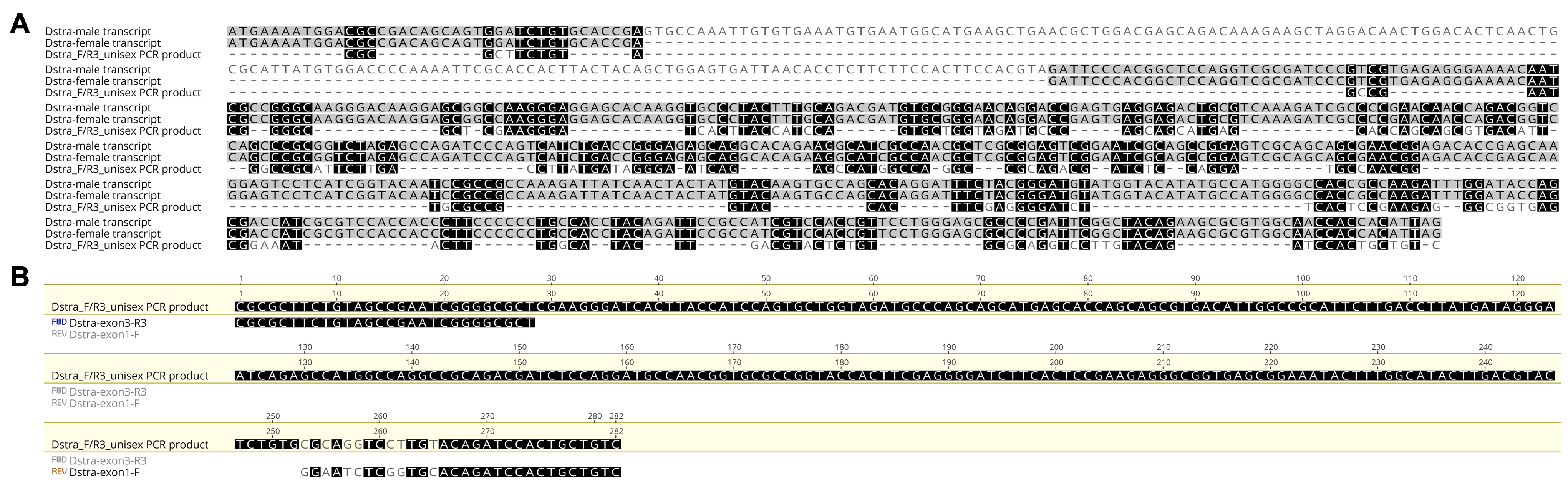

Supplement: Supplementary file 3 — Fig. S3 Verification of the unisex polymerase chain reaction (PCR) product from the reverse‐transcript (RT)‐PCR against Dstra gene. (A) Alignment of the Dstra male transcript, female transcript, and the unisex PCR product (Multiple Alignment using Geneious software). Low similarities were identified between the unisex product and the Dstra male (27%) or female (34%) transcript. (B) Alignment of the unisex PCR product and corresponding primer sequences (Map to Reference using Geneious software), indicating that this unisex product is a nonspecific amplification of the corresponding primers. [file INS-33-547-s001.jpg]

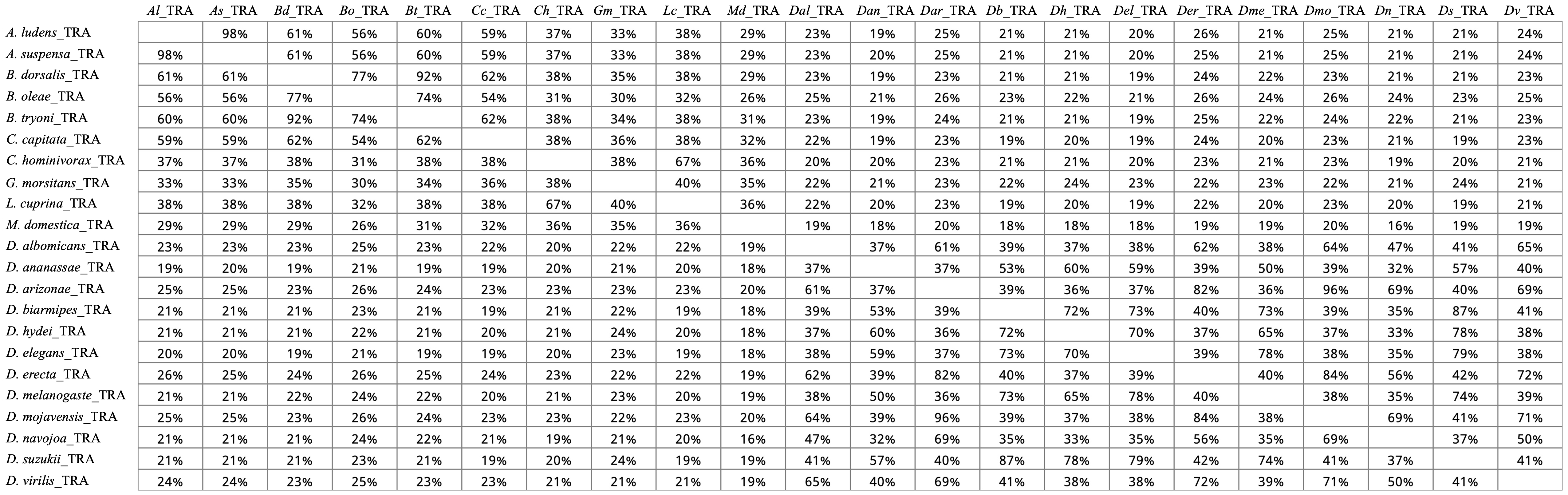

Supplement: Supplementary file 4 — Fig. S4 Distance matrix of TRANSFORMER (TRA) proteins from different Dipteran species. See Fig. 2 legend for the sequence identifiers and alignment methods. [file INS-33-547-s004.jpg]

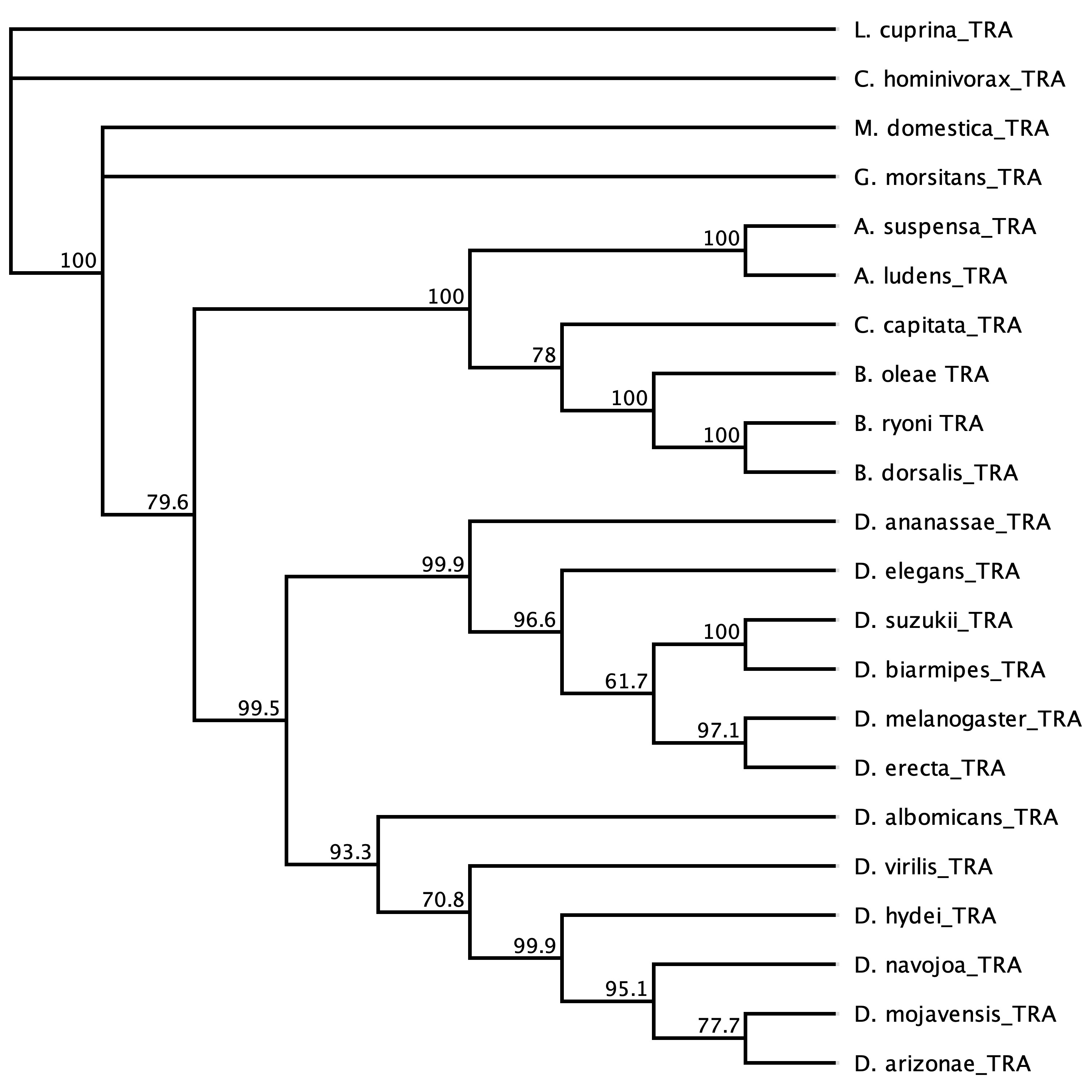

Supplement: Supplementary file 5 — Fig. S5 Phylogenetic analysis of dipteran TRANSFORMER (TRA) proteins. Unrooted neighbor‐joining tree was constructed using TRA amino acid sequences. Bootstrap values (1 000 replicates) are shown on the nodes of the trees. See Fig. 2 legend for the sequence identifiers and alignment methods. [file INS-33-547-s006.jpg]

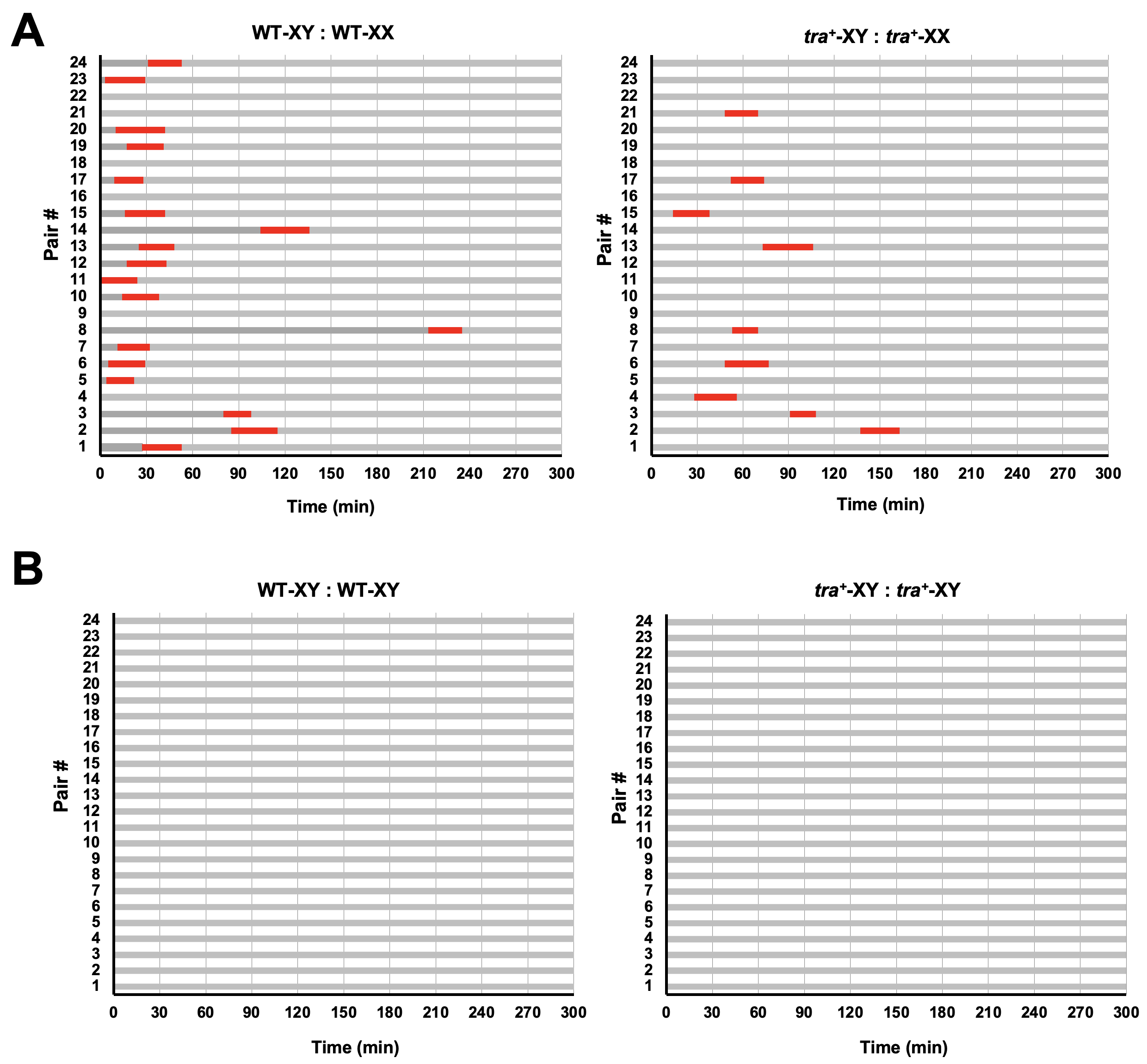

Supplement: Supplementary file 6 — Fig. S6 Analysis for copulation success. Twenty‐four pairs of genetic male and female (A) and 24 pairs of genetic males (B) were recorded for 300 min. The red bar indicates the copulation period, whereas the gray bar indicates no copulation. The genotypes of the flies used for analysis are indicated. [file INS-33-547-s005.jpg]
