# Supplementary material for: The transformer gene controls sexual development in Drosophila suzukii
Source: Insect Sci. 2025 Mar 30;33(2):547–65. doi: 10.1111/1744-7917.70031 (PMC13087859; doi:10.1111/1744-7917.70031)
Supplement: Supplementary file 7 — Table S1 Primer sequences [file INS-33-547-s003.docx]

**Table S1** Primer sequences.

| Primer No. | Primer name | Primer sequence |  |
| --- | --- | --- | --- |
| P86 | Dstra_Gene_R | ACTTCCACTTCCTAACTCGTGTGACC | |
| P88 | Dstra_Gene_F | AGGCATGGGAGGAAAAGGAAGAACT | |
| P85 | Dstra_mRNA_F | CTCTGAAAATGGACGCCGACAG | |
| P87 | Dstra_mRNA_R | TGTAATTATGTCGAATGTTGTCTGTC | |
| P692 | Dstra_XhoI-MluI_F | AAATCTCGAGAACGCGTATGAAAATGGACGCCGACAGCAGTGGATCTGTGCA | |
| P694 | Dstra_AvrII_R | AAATCCTAGGCTAGGCTAATGTGGTGGTTGCCACGCGCTTCTCCCA | |
| P695 | Dstra_HindIII-BsmBI_F | GTTGAAGCTTGCGCCCTCTGCATGAAAATGGACGCCGACAGCAGTGGATCTGTGCA | |
| P696 | Dstra_NheI_R | GTTGGCTAGCCTAGGCTAATGTGGTGGTTGCCACGCGCTTCTCCCA | |
| P_742 | Dstra_exon1-F1 | GACAGCAGTGGATCTGTGCACCGAGATTCC | |
| P_703 | Dstra_exon2-R1 | GCCTGCTCTCCCGGTCAGATGACTGGGATCT | |
| P_704 | Dstra_exon2-R2 | CCTGTGCTGGCACTTGTACATAGTAGTTGATAATCTT | |
| P_705 | Dstra_exon3-R3 | CGCGCTTCTGTAGCCGAATCGGGGCGCT | |
| P1453 | Dsβ2t_F1 | ATGCGTGAAATCGTGCACATTCAGGCCGGA | |
| P1454 | Dsβ2t_R1 | ATCACTGTCCCCGTAATACGTTCCGG | |
| P1358 | DsYp1_F | CTGCAGCAAGTCTACCACCTG | |
| P1359 | DsYp1_R | GCGCTGAATCATCTCGTTCAG | |
| P740 | DsTBP_F | CCACGGTGAATCTGTGCT | |
| P741 | DsTBP_R | GGAGTCGTCCTCGCTCTT | |
| P420 | AmCyan_F | CCTGTCCAACAAGTTCATCGG | |
| P516 | AmCyan_R | CAGCCGGTGGTCTTCTTGG | |
| MFS5 | TREhs43_F | AGGCGCTTCGTCTACGGA | |
| MFS10 | 5’pBac_R | ACGACCGCGTGAGTCAAAATGACG | |
| P2215 | Dsfru_P1M_F | GTACGCCCTCTTCCGCGGTCCG | |
| P2216 | Dsfru_fsExon_F | GGCATAAACCGAGCACCAAGGCG | |
| P2261 | Dsfru_C2Exon_R | GGGATGTGGATGCTGGTTCTGTAGG | |
| P2259 | Dsdsx_E3_F | TGCAGACGCCAATATTGAAGAGGC | |
| P2179 | Dsdsx_msE5_R | GTCGGCGGACAAATCTGGGTGA | |
| P2180 | Dsdsx_fsE4_R | CCGGCGGTCACACATCGACATA | |
